# Supplementary material for: A Distinct Class of Genome Rearrangements Driven by Heterologous Recombination
Source: Mol Cell. 2018 Jan 18;69(2):292–305.e6. doi: 10.1016/j.molcel.2017.12.014 (PMC5783719; doi:10.1016/j.molcel.2017.12.014)
Supplement: Document S1. Figures S1–S4 and Table S1 [file mmc1.pdf]

Molecular Cell, Volume *69*

Supplemental Information

A Distinct Class of Genome Rearrangements

Driven by Heterologous Recombination

Ana María León-Ortiz, Stephanie Panier, Grzegorz Sarek, Jean-Baptiste Vannier, Harshil Patel, Peter J. Campbell, and Simon J. Boulton

SUPPLEMENTAL DATA

A Distinct Class of Genome Rearrangements Driven by Heterologous Recombination

Ana María León-Ortiz[1], Stephanie Panier[1], Grzegorz Sarek[1], Jean-Baptiste Vannier[2], Harshil Patel[3], Peter J. Campbell[4] & Simon J. Boulton[1,*]

Supplemental Figure S1. Visual method to score Ht-REC in *Caenorhabditis elegans.*
Supplemental Figure S2. Scoring Ht-REC using a second set of genetic markers.
Supplemental Figure S3. Effect of mutating msh-2/Msh2 on rtel-1/Rtel1 mutant phenotypes.
Supplemental Figure S4. Generating Msh2 CRISPR/Cas9 knockout cell lines.
Supplemental Table S1. Scoring heterologous recombination.
Supplemental Table S2. SVs in Rtel1-deficient subclones (*separate excel file*).
Supplemental Table S3. SVs in wild-type subclones (*separate excel file*).

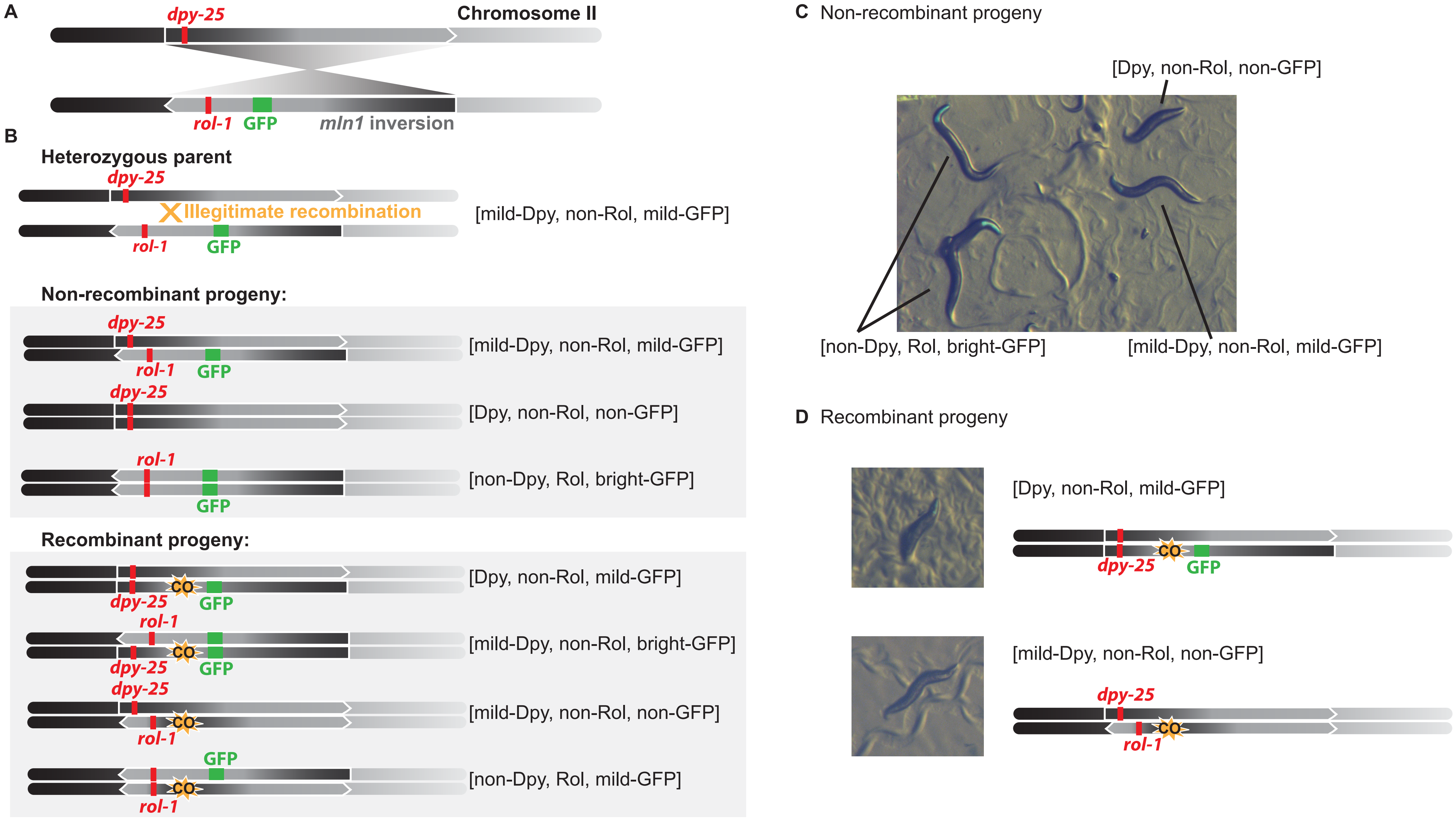

Supplemental Figure S1. Visual method to score Ht-REC in *Caenorbabditis elegans*. Related to Figures 1, 2 and 3.

(A) The system used to score heterologous recombination in *C. elegans* relies on the *mIn1* inversion on chromosome II and visible genetic marker. One copy of chromosome II (top) is marked with the semi-dominant *dpy-25* mutation. The second copy of chromosome II (bottom) contains the *mIn1* inversion marked with the recessive *rol-1* mutation, as well as a semi-dominant insertion of a GFP-expressing transgene.

(B) Recombination is scored in a heterozygous parent that carries one normal copy of chromosome II and one copy that has the *t* inversion. Chromosome organisation and the corresponding phenotypes expected are presented for non-recombinant and for recombinant progeny. CO: crossover location resulting from illegitimate recombination. Note that not all possible recombinant progeny is depicted.

(C-D) Representative images of non-recombinant (C) and recombinant (D) worms. The corresponding phenotypes are indicated for each worm and the diagrams represent a possible crossover event that may explain the phenotypes observed in these worms.

A Heterozygous parent

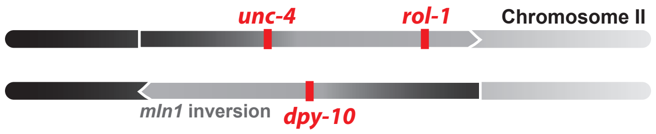

B

	Non-recombinant			Recombinant				
	non-Unc non-Rol non-Dpy	Unc Rol non-Dpy	non-Unc non-Rol Dpy	Unc non-Rol Dpy	non-Unc Rol non-Dpy	non-Unc Rol Dpy	non-Unc Rol non-Dpy	Recombinant / Total (Percent)
WT	2253	826	826	0	0	2	0	2 / 3907 (0.05)
rtel-1	1927	701	980	3	0	46	3	52 / 3660 (1.4)

Supplemental Figure S2. Scoring Ht-REC using a second set of genetic markers. Related to STAR Methods.
(A) Diagram representing the second system used to score heterologous recombination. A heterozygous parent carries one normal chromosome II, marked with recessive *unc-4* and *rol-1* mutations, and one chromosome II with the *mIn1* inversion marked with a recessive *dpy-10* mutation. These parental worms are [non-Unc, non-Rol, non-Dpy].
(B) Table showing the non-recombinant and recombinant progeny scored using the system described in (A) in wild-type and *rtel-1* mutant backgrounds.

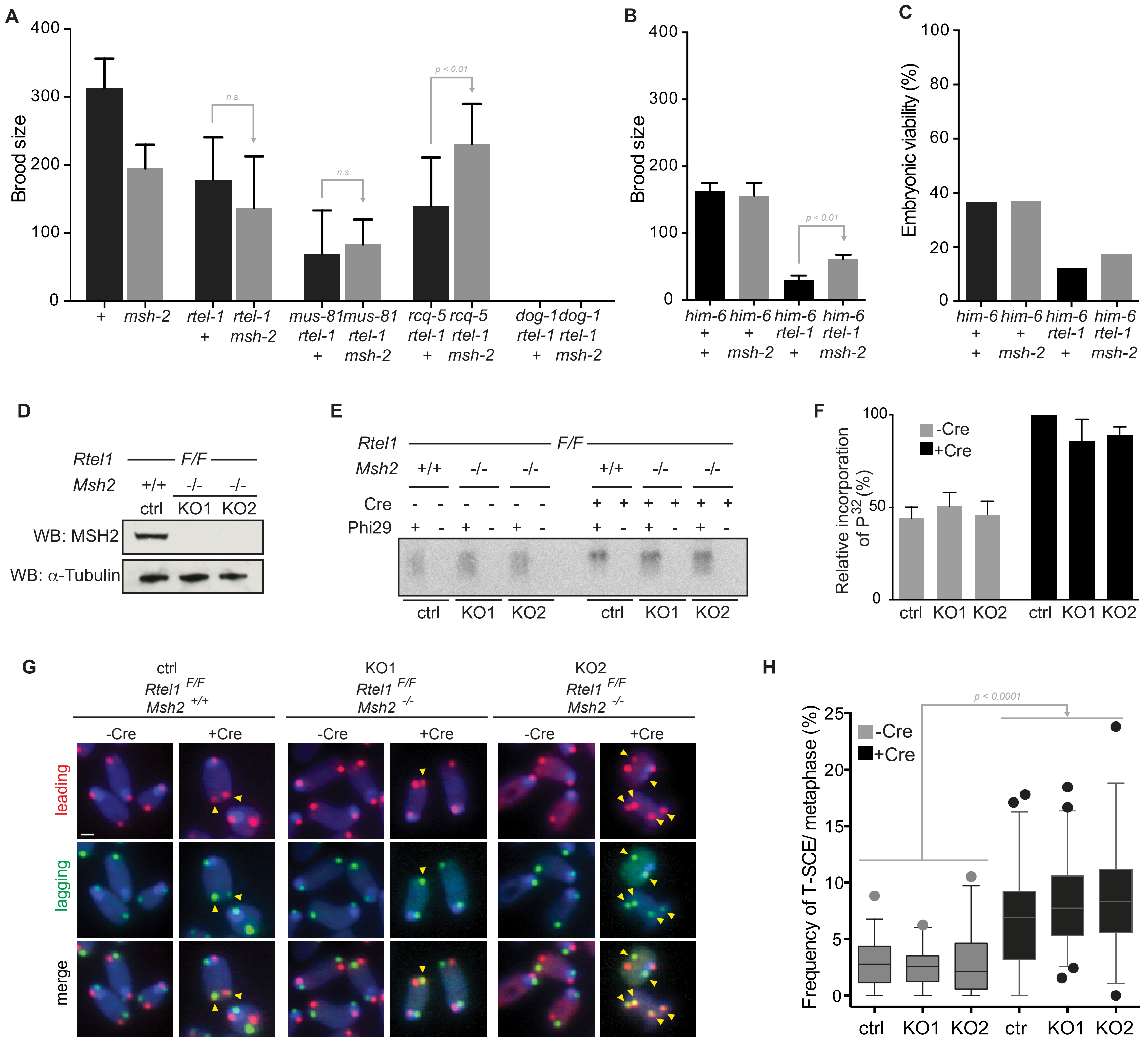

A
B
C
D
E
F
G
H
Brood size
Embryonic viability (%)
n.s.
p < 0.01
+
msh-2
rtel-1
mus-81
rcq-5
dog-1
him-6
Rtel1
F/F
Msh2
+/+
-/-
ctrl
KO1
KO2
WB: MSH2
WB: α-Tubulin
Cre
Phi29
Relative incorporation of P32 (%)
-Cre
+Cre
leading
lagging
merge
p < 0.0001
Frequency of T-SCE/ metaphase (%)
ctr

Supplemental Figure S3. Effect of mutating *msh-2*/*Msh2* on *rtel-1*/*Rtel1* mutant phenotypes. Related to Figure 4.

(A-B) Brood sizes of the indicated genotypes. The brood size of at least 12 worms was scored for each genotype. Error bars correspond to standard error of the mean. Statistics: Mann-Whitney Wilcoxon test.

(C) Percentage of viable embryos for worms of the indicated genotypes. For each genotype, at least 700 embryos were scored for viability.

(D) Western blot showing loss of Msh2 expression in the CRISPR/Cas9 knockout cell lines KO1 and KO2.

(E-F) T-circle amplification assay upon Cre-driven excision of *Rtel1* in the control or the *Msh2* knockout cell lines (KO1 and KO2). (E) shows a representative image of the assay. Three independent experiments were quantified in (F).

(G-H) Telomere sister-chromatid exchanges visualized by chromosome-oriented FISH on metaphase spreads. Two telomeric probes specific to the leading (green) or the lagging (red) strand were used. Yellow arrowheads indicate overlap between the green and the red signal where recombination between telomeres has taken place (G). Scale bar: 10μm. Two independent experiements were quantified in (H). The graph represents a box and whiskers plot where the whiskers correspond to the 5th-95th

A

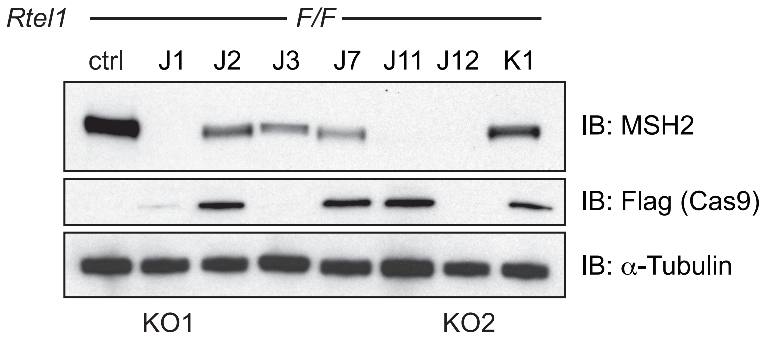

B

Guide RNA

```
WT    TAAGGAGACGCTGCAGTTGGAAGGCGCGGCCGAGGCGGGCTTCGTGCGCTTCTTTGAGGGCATG

KO1   TAAGGAGACGCTGCAGT----------------------------GCGCTTCTTTGAGGGCATG   9/10   STOP codon exon 1
      TAAGGAGACGCTGCAGT----------------------------GCGCTTCTTCGAGGGCATG   1/10   STOP codon exon 1

KO2   TAAGGAGACGCTGCAGTTGGAAGGC-----------GGGCTTCGTGCGCTTCTTTGAGGGCATG   4/10   STOP codon exon 1
      TAAGGAGACGCTGCAGTTGGAAGG-----------TGGGCTTCGTGCGCTTCTTTGAGGGCATG   1/10   STOP codon exon 1
      TAAGGAGACGCTGCAGTTGGAAGGCGCGGC-GAGGCGGGCTTCGTGCGCTTCTTTGAGGGCATG   5/10   STOP codon exon 1
```

Supplemental Figure S4. Generating Msh2 CRISPR/Cas9 knockout cell lines. Related to Figure S3D and STAR Methods.

(A) Western-blot showing the screening of clonal populations for complete loss of the MSH2 protein. The two knockout cell lines used in this study (labelled KO1 and KO2 under the blots) were chosen on the basis of the low or absent expression of Cas9 (revealed by a Flag antibody).

(B) DNA sequences of the two *Msh2* knockout cell lines used in this study. The sequence targeted by the guide RNA used is shown.

	Non-recombinant progeny (percent)			Recombinant progeny (percent)					
Genotype	Mild-Dpy Mild-GFP Non-Roller	Dpy Non-GFP Non-Roller	Non-Dpy Bright-GFP Roller	Dpy GFP Non-Roller	Mild-Dpy Bright-GFP Non-Roller	Non-Dpy Non-GFP	Mild-GFP Roller	Non-Dpy Mild-GFP Non-Roller	Recombinants / Total (percent)
WT	1726 (54.4)	600 (18.9)	847 (26.7)	2 (0.06)	0 (0)	0 (0)	0 (0)	0 (0)	2/3175 (0.06)
rtel-1	1533 (58.4)	410 (15.6)	639 (24.3)	35 (1.30)	0 (0)	8 (0.30)	0 (0)	0 (0)	43/2625 (1.6)
lig-4	897 (58.8)	219 (14.4)	401 (26.3)	7 (0.46)	0 (0)	1 (0.07)	0 (0)	0 (0)	8/1525 (0.52)
polq-1	1315 (58.0)	336 (14.8)	608 (26.8)	8 (0.35)	0 (0)	0 (0)	0 (0)	0 (0)	8/2267 (0.35)
him-6	467 (49.2)	116 (12.2)	303 (31.9)	63 (6.60)	0 (0)	0 (0)	0 (0)	0 (0)	63/949 (6.6)
rcq-5	1776 (54.3)	623 (19)	867 (26.5)	0 (0)	0 (0)	2 (0.06)	0 (0)	0 (0)	2/3268 (0.06)
dog-1	1259 (57)	415 (18.8)	537 (24.2)	1 (0.05)	0 (0)	0 (0)	0 (0)	0 (0)	1/2212 (0.05)
brc-1	1147 (59.6)	435 (22.6)	306 (15.9)	31 (1.60)	0 (0)	0 (0)	6 (0.31)	0 (0)	37/1925 (1.9)
msh-2	1178 (54.4)	372 (17.7)	612 (28.3)	3 (0.14)	0 (0)	0 (0)	1 (0.05)	0 (0)	4/2166 (0.18)
msh-6	1040 (55)	384 (20.3)	462 (24.4)	5 (0.27)	0 (0)	1 (0.53)	0 (0)	0 (0)	6/1892 (0.32)
mlh-1	696 (55.1)	263 (20.8)	301 (23.9)	3 (0.24)	1 (0.08)	0 (0)	0 (0)	0 (0)	4/1264 (0.32)
pms-2	600 (56.6)	171 (16.1)	287 (27)	3 (0.28)	0 (0)	0 (0)	0 (0)	0 (0)	3/1061 (0.28)
rtel-1; lig-4	652 (57.1)	162 (14.2)	296 (25.9)	31 (2.7)	0 (0)	0 (0)	0 (0)	0 (0)	31/1141 (2.7)
rtel-1; polq-1	241 (60.25)	64 (16)	83 (20.75)	12 (3.0)	0 (0)	0 (0)	0 (0)	0 (0)	12/400 (3.0)
msh-2 rtel-1	673 (51.3)	312 (23.8)	322 (24.6)	4 (0.30)	0 (0)	0 (0)	0 (0)	0 (0)	4/1311 (0.31)
msh-2; him-6	240 (59.7)	30 (7.5)	98 (24.4)	29 (7.20)	2 (0.5)	0 (0)	3 (0.75)	0 (0)	34/402 (8.46)
msh-2; brc-1	1029 (54.2)	405 (21.3)	463 (24.4)	1 (0.05)	1 (0.05)	0 (0)	0 (0)	0 (0)	2/1899 (0.11)
msh-6 rtel-1	543 (57)	157 (16.5)	247 (26)	4 (0.42)	1 (0.10)	0 (0)	1 (0.10)	0 (0)	6/953 (0.63)
rtel-1; mlh-1	196 (55.8)	60 (17.1)	73 (20.8)	22 (6.28)	0 (0)	0 (0)	0 (0)	0 (0)	6/351 (6.27)
mlh-1; him-6	329 (56.5)	59 (10)	119 (20.5)	54 (9.5)	6 (1)	0 (0)	0 (0)	3 (0.5)	73/580 (12.6)
rtel-1; pms-2	470 (54)	161 (18.5)	227 (26.1)	12 (1.4)	0 (0)	0 (0)	0 (0)	0 (0)	12/870 (1.4)

Reciprocal exchange events

Supplemental Table S1. Scoring heterologous recombination. Related to Figures 1, 2 and 3.

Numbers of non-recombinant and recombinant progeny of each phenotype in the indicated genetic backgrounds.
