# Supplementary material for: A Distinct Class of Genome Rearrangements Driven by Heterologous Recombination
Source: Mol Cell. 2018 Jan 18;69(2):292–305.e6. doi: 10.1016/j.molcel.2017.12.014 (PMC5783719; doi:10.1016/j.molcel.2017.12.014)
Supplement: Document S2. Article plus Supplemental Information [file mmc4.pdf]

Molecular Cell

Article

A Distinct Class of Genome Rearrangements Driven by Heterologous Recombination

Graphical Abstract

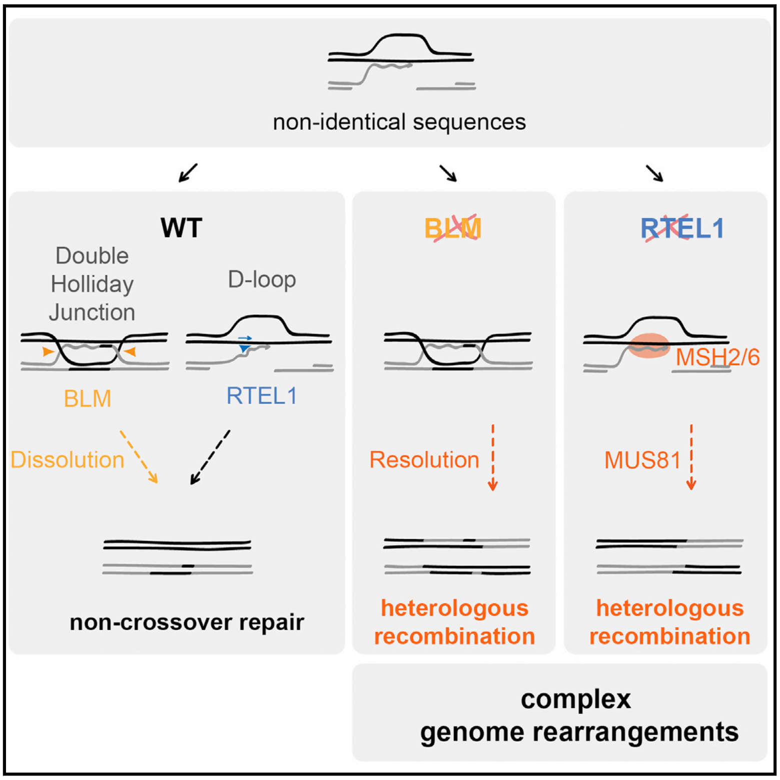

Highlights

- Heterologous recombination (Ht-REC) can occur between non-identical sequences
- RTEL1, HIM-6/BLM, and BRC-1/BRCA1 prevent Ht-REC
- MSH-2/MSH-6 drives Ht-REC in the absence of RTEL-1 or BRC-1
- Lack of mammalian RTEL1 causes complex genome rearrangements consistent with Ht-REC

Authors

Ana María León-Ortiz, Stephanie Panier, Grzegorz Sarek, Jean-Baptiste Vannier, Harshil Patel, Peter J. Campbell, Simon J. Boulton

Correspondence

simon.boulton@crick.ac.uk

In Brief

León-Ortiz et al. demonstrate that recombination is possible between non-identical sequences (Ht-REC) but is blocked by RTEL-1, HIM-6/BLM, and BRC-1/BRCA1 in *C. elegans*. MSH-2/MSH-6 instead drives Ht-REC in the absence of RTEL-1 or BRC-1. Ht-REC likely contributes to complex genome rearrangements in the absence of mammalian RTEL1 and in cancer.

León-Ortiz et al., 2018, Molecular Cell *69*, 292–305
January 18, 2018 © 2017 The Author(s). Published by Elsevier Inc.
https://doi.org/10.1016/j.molcel.2017.12.014

A Distinct Class of Genome Rearrangements Driven by Heterologous Recombination

Ana María León-Ortiz,[1] Stephanie Panier,[1] Grzegorz Sarek,[1] Jean-Baptiste Vannier,[2] Harshil Patel,[3] Peter J. Campbell,[4] and Simon J. Boulton[1,5,*]
[1]DSB Repair Metabolism Laboratory, The Francis Crick Institute, 1 Midland Road, London NW1 1AT, UK
[2]Telomere Replication and Stability Group, MRC London Institute of Medical Sciences, Faculty of Medicine, Imperial College London, Hammersmith Hospital Campus, London W12 0NN, UK
[3]Bioinformatics and Biostatistics, The Francis Crick Institute, 1 Midland Road, London NW1 1AT, UK
[4]Wellcome Trust Sanger Institute, Wellcome Trust Genome Campus, Hinxton, Cambridge CB10 1SA, UK
[5]Lead Contact
*Correspondence: simon.boulton@crick.ac.uk
https://doi.org/10.1016/j.molcel.2017.12.014

SUMMARY

Erroneous DNA repair by heterologous recombination (Ht-REC) is a potential threat to genome stability, but evidence supporting its prevalence is lacking. Here we demonstrate that recombination is possible between heterologous sequences and that it is a source of chromosomal alterations in mitotic and meiotic cells. Mechanistically, we find that the RTEL1 and HIM-6/BLM helicases and the BRCA1 homolog BRC-1 counteract Ht-REC in *Caenorhabditis elegans*, whereas mismatch repair does not. Instead, MSH-2/6 drives Ht-REC events in *rtel-1* and *brc-1* mutants and excessive crossovers in *rtel-1* mutant meioses. Loss of vertebrate *Rtel1* also causes a variety of unusually large and complex structural variations, including chromothripsis, breakage-fusion-bridge events, and tandem duplications with distant intra-chromosomal insertions, whose structure are consistent with a role for RTEL1 in preventing Ht-REC during break-induced replication. Our data establish Ht-REC as an unappreciated source of genome instability that underpins a novel class of complex genome rearrangements that likely arise during replication stress.

INTRODUCTION

Genome instability is the driving force that causes mutations and chromosome rearrangements, which ultimately lead to the development of cancers. Chromosomal translocations that result in gene fusions have been recognized for many decades as drivers of tumor development. Many of these rearrangements occur between sequences that share no homology and are believed to occur via non-homologous end joining (NHEJ), in which chromosomal breaks from different parts of the genome are joined together by simple ligation (Bunting and Nussenzweig, 2013). In addition, a significant number of rearrangements contain microhomologies at the breakpoints, which have been proposed to arise by microhomology-mediated end joining (MMEJ) (Sfeir and Symington, 2015). However, because MMEJ shares the same initial double-strand break (DSB) resection step with homologous recombination (HR) (Truong et al., 2013), it is also possible that these rearrangements could occur via heterologous recombination resulting from erroneous strand invasion between sequences with limited homology.

HR is typically an error-free mechanism that ensures the accurate repair of DSBs and allows the restart of stalled replication forks. In meiotic cells, HR repairs programmed DSBs, creating a physical link between homologous chromosomes that is essential for their segregation at the first meiotic division while also generating genetic diversity. The current model for HR dictates that the 5′ end of a DSB is resected to produce a 3′ overhang that, when bound by the Rad51 recombinase, is used to search the genome for a homologous sequence to invade. Upon invasion, a displacement loop (D-loop) is formed, in which the invading 3′ end is extended by DNA synthesis. If it is then displaced, it can anneal with the second end of the break, and repair is achieved by DNA synthesis and ligation, which is termed synthesis-dependent strand annealing (SDSA). Evidence suggests that the D-loop-disrupting activity of the helicase RTEL1 is important for promoting this mechanism (Barber et al., 2008; Vannier et al., 2012, 2013; Youds et al., 2010). SDSA does not result in exchange of the chromosomal arms between the two DNA molecules involved and, therefore, produces exclusively non-crossovers (NCOs). If, instead, the second end of the break is captured by the D-loop, this can lead to the formation of a double Holliday junction (dHJ), which is either dissolved to produce exclusively NCO events or resolved to produce both crossovers (COs) and NCOs. Although dissolution is achieved by the BTR complex, composed of the BLM helicase, the TOP3 topoisomerase, and an RMI scaffold (RMI1 and 2) (Mankouri and Hickson, 2007; Wu and Hickson, 2003; Xu et al., 2008), resolution involves the action of several different structure-specific endonucleases that can cleave the junctions to result in either CO or NCO products (for reviews, see Jasin and Rothstein, 2013; West et al., 2015).

 © 2017 The Author(s). Published by Elsevier Inc.
This is an open access article under the CC BY license (http://creativecommons.org/licenses/by/4.0/).

An important step in controlling the quality of HR occurs at the level of template choice. In mitotically dividing cells, one strategy to achieve this is to limit recombination to the S and G2 phases of the cell cycle, where an identical DNA template is available on the sister chromatid (Chapman et al., 2012; Daley and Sung, 2014). Nevertheless, recombination between sequences that are similar but not identical remains possible, and avoiding these events has been shown in prokaryotes and simple eukaryotes to require different DNA repair factors. This type of recombination is termed homeologous recombination, and genes in the mismatch repair (MMR) pathway, together with certain helicases and nucleases, are known to play an important role in suppressing these events. The precise mechanism by which MMR proteins suppress homeologous recombination remains unclear, but studies in bacteria suggest that MutS, MutL, and UvrD act to interfere with homeologous strand exchange and/or branch migration (Alani et al., 1994; Detloff et al., 1992; Tham et al., 2013; Westmoreland et al., 1997; Worth et al., 1994, 1998). In budding yeast, *MSH2*, *MSH3*, *MSH6*, *MLH1*, and *PMS1* have been implicated in suppressing homeologous recombination in mitotic cells (Datta et al., 1996; Nicholson et al., 2000; Selva et al., 1995), whereas *MSH2* and *PMS1* have a similar role during meiosis (Chambers et al., 1996; Hunter et al., 1996). In addition, the UvrD homolog *SRS2*, the *BLM* homolog *SGS1*, and *MPH1* are also involved in this process (Myung et al., 2001; Spell and Jinks-Robertson, 2004; Tay et al., 2010; Welz-Voegele and Jinks-Robertson, 2008). In mice, *Msh2* is required to prevent recombination between homeologous sequences in embryonic stem cells (de Wind et al., 1995; Larocque and Jasin, 2010), but no helicase has been identified in metazoans with such an activity, and, importantly, BLM is not required in mouse or human cells to prevent homeologous recombination (Larocque and Jasin, 2010).

Using a reporter system in *Caenorhabditis elegans* to detect heterologous recombination (Ht-REC), we demonstrate that recombination between heterologous sequences is extremely rare in wild-type animals but does occur with high frequency in mutants defective for *C. elegans* RTEL1, BRCA1, and BLM (RTEL-1, BRC-1, and HIM-6, respectively). Strikingly, we find that deletion of the MSH-2/6 complex, but not other MMR genes, completely suppresses all Ht-REC events that occur in *rtel-1* or *brc-1* mutants, whereas the illegitimate COs in *him-6* are unaffected. We proceed to establish that the excessive meiotic CO events that occur in *rtel-1* mutants are also alleviated by *msh-2* deletion. Whole-genome sequencing of vertebrate cells lacking RTEL1 also revealed a significant accumulation of unusually large and complex structural variations, including chromothripsis and breakage-fusion-bridge events frequently observed in cancer genomes, as well as a novel class of events involving tandem duplication with distant intra-chromosomal insertion. Importantly, many of the structural variations that arise in the absence of *Rtel1* are associated with an increase in copy number, arguing for Ht-REC occurring during DNA replication. Collectively, our study establishes the existence of atypical classes of genome rearrangements driven by Ht-REC that likely contribute to the complex rearrangements prevalent in cancer genomes.

RESULTS

RTEL-1 Limits Erroneous Crossover Events

Because of their ability to prevent recombination across a given chromosomal interval, genetic balancers have been used both in *Drosophila melanogaster* and *C. elegans* genetics to stably maintain, as heterozygous, mutations that cause sterility or lethality when they are homozygous. Although meiotic breaks are induced both along balancers and the chromosome with which they are paired, and despite inhibition of inter-sister repair during early meiosis, crossing over is extremely rare within a balancer, most likely because of the heterology in sequence with the paired chromosome (Beadle and Sturtevant, 1935; Novitski and Braver, 1954; Zetka and Rose, 1992).

We used the 8-Mbp *mIn1* inversion on chromosome II to assay heterologous recombination (Edgley and Riddle, 2001) by scoring the exchange of genetic markers flanking part of the inversion (Figure 1A; Figure S1; STAR Methods). In wild-type worms, only 2 of 3,175 progenies exhibited an exchange of flanking markers consistent with erroneous repair within the *mIn1* inversion, showing that these events are extremely rare (0.06% of the total progeny; Figure 1B; Table S1) and confirming that *mIn1* is refractory to meiotic recombination.

Because the RTEL1 helicase disassembles D-loops, we reasoned that it could act to ensure the quality of meiotic recombination by dismantling erroneous strand-pairing events arising from the invasion of a broken DNA end into a donor sequence with limited homology, such as within the context of a balanced region. Indeed, in the absence of RTEL-1, we recovered 43 worms of 2,625 progenies displaying an exchange of flanking markers (1.64%; Figure 1B; Table S1) within the *mIn1* inversion, which corresponds to a 27-fold increase in Ht-REC events over the wild-type. This number is likely an underestimation because products of reciprocal exchange events should appear in similar proportions but they do not because of the effect of certain recombinant genotypes on viability (Table S1). Furthermore, potential double crossover events will not result in exchange of flanking markers and will, therefore, not score in this system. We also find a similar increase in the rate of flanking marker exchange in *rtel-1* mutant worms using different sets of genetic markers on the *mIn1* inversion (Figure S2; STAR Methods).

To exclude the possibility that the erroneous repair events in *rtel-1* result from ligation of meiotic DSBs by either NHEJ or MMEJ, we inactivated these repair pathways by mutating *lig-4* or *polq-1*, respectively. As single mutants, *lig-4* and *polq-1* exhibited an intermediate increase in flanking marker exchange within the *mIn1* inversion (Figure 1B), suggesting that loss of these mechanisms favors erroneous repair to some extent, as seen in other systems (Pierce et al., 2001; Zelensky et al., 2017). More importantly, mutation of *lig-4* or *polq-1* in an *rtel-1* background did not prevent the occurrence of genetic marker exchange (Figure 1B), demonstrating that these events do not arise as a result of NHEJ or MMEJ.

Because the *mIn1* sequence we use is an internal inversion, it is conceivable that the central part of the chromosome could re-orient in a way that allows its homologous alignment to produce gene conversion between homologously paired sequences. If this did occur, the resulting worms should carry no

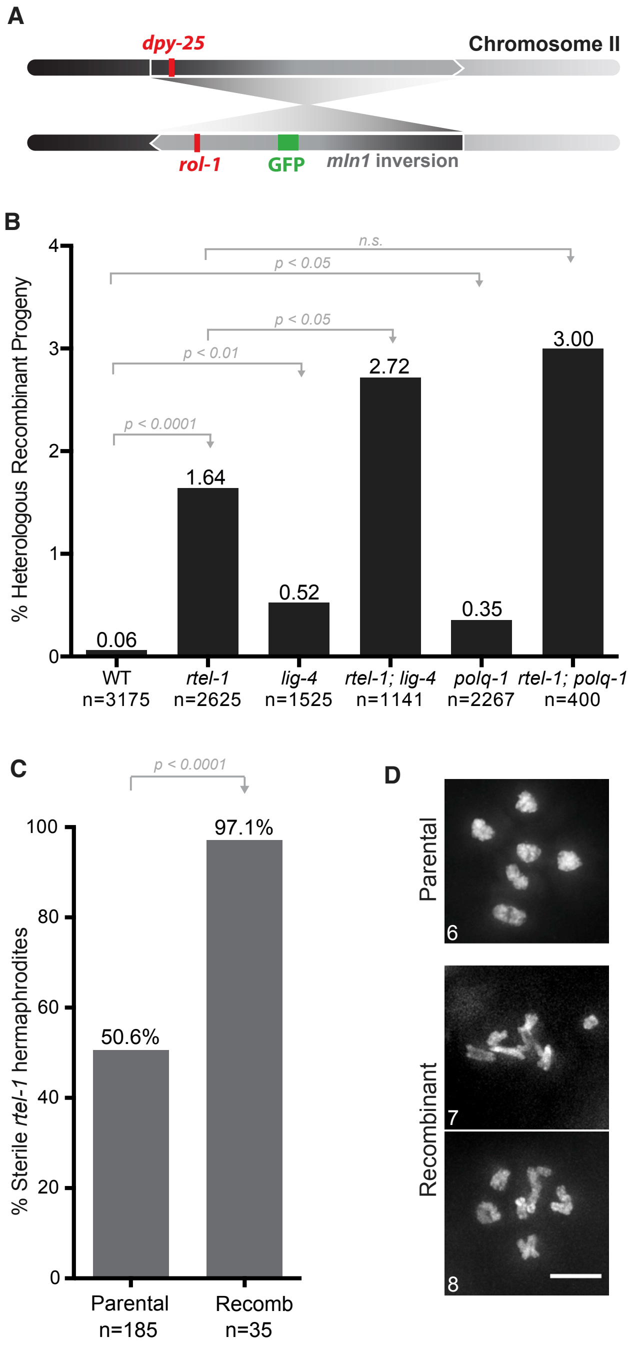

Figure 1. RTEL-1 Prevents Recombination between Heterologous Sequences

(A) The system used to score heterologous recombination in *C. elegans* relies on the use of the *mIn1* inversion on chromosome II. Animals in which recombination is scored contain one normal chromosome II (top), whereas the second copy carries the *mIn1* inversion (bottom). Genetic markers flanking the inversion are shown.

(B) Percentage of heterologous recombinant progeny. n, number of worms scored. Statistics: χ^2 tests.

(C) Percentage of sterile worms among the *rtel-1* parental hermaphrodites and the *rtel-1* recombinant hermaphrodites. Statistics: Fisher's exact test.

(D) DAPI-stained chromosomes at diakinesis in a parental hermaphrodite or two recombinant hermaphrodites (F1). The number of DAPI-stained bodies detectable through the z stack is indicated on each image. Scale bar, 5 μm. See also Table S1.

other modification than a gene conversion tract. In contrast, recombinant worms produced by Ht-REC will contain segmental aneuploidies as a result of loss of a portion of either the inversion or the heterologous chromosome (Figures S1B and S1D). Consistent with the latter, we find that 97.1% of the recombinant worms are sterile, in contrast to 50.6% of the parental worms (Figure 1C). Furthermore, although the diakineses of parental hermaphrodites present 6 DAPI-stained bivalents, as seen in wild-type worms, diakineses of recombinant F1 worms present aberrantly shaped chromosomal bodies as well as interlinked DAPI-stained material that cannot be clearly distinguished into 6 independent bivalents, suggesting ongoing genetic instability and an accumulation of abnormal chromosome structures (Figure 1D). Collectively, our results argue that the erroneous events observed in *rtel-1* represent Ht-REC events and occur independently of NHEJ and MMEJ.

HIM-6/BLM and BRC-1/BRCA1 Also Limit Illegitimate Crossover Events

We next examined helicase mutants that are synthetic lethal in combination with *rtel-1* (Barber et al., 2008) because redundancy in preventing Ht-REC events might be the underlying cause of these genetic interactions. We therefore examined *him-6*, *dog-1*, and *rcq-5* mutants, which are the homologs of human BLM, FANCJ and RECQ5, respectively (Hu et al., 2007). Mutation of *him-6/BLM*, but not *dog-1/FANCJ* or *rcq-5*, resulted in a dramatic increase in Ht-REC events within the *mIn1* inversion (Figure 2A; Table S1). Similar to *rtel-1* mutants, *him-6* recombinant worms are sterile, suggesting that they also carry segmental aneuploidies (Figure 2B). Importantly, *him-6* mutants in *C. elegans* show a decrease in the level of normal meiotic CO (Wicky et al., 2004), whereas the levels of flanking marker exchange within the inversion are extremely high (6.64%, a 110-fold increase versus the wild-type [WT]), which suggests that the role of HIM-6 in preventing Ht-REC is distinct from its function in normal meiosis.

Because meiotic DSBs generated within balanced regions cannot be repaired through the paired chromosome because of a lack of local sequence homology, they are instead repaired late in meiosis using the sister chromatid as a template. We therefore examined the effect of blocking inter-sister repair by mutating the BRCA1 homolog, *brc-1* (Adamo et al., 2008), and observed an increase of 1.92% in Ht-REC events in this mutant (Figure 2C; Table S1). The sterility of the *brc-1* recombinant F1 worms is greatly increased with respect to that of the parental line, consistent with the presence of segmental aneuploidies (Figure 2D). This suggests that, when inter-sister repair is compromised, meiotic DSBs within balanced heterologous sequences are forced to use the heterologous chromosome as a repair template.

The occurrence of Ht-REC in *brc-1* mutants raised the possibility that RTEL-1 and/or HIM-6/BLM could also be involved in inter-sister repair. We assessed the integrity of inter-sister repair in both of these mutants, in the absence of heterologous sequences, by combining them with *syp-2* mutants, in which

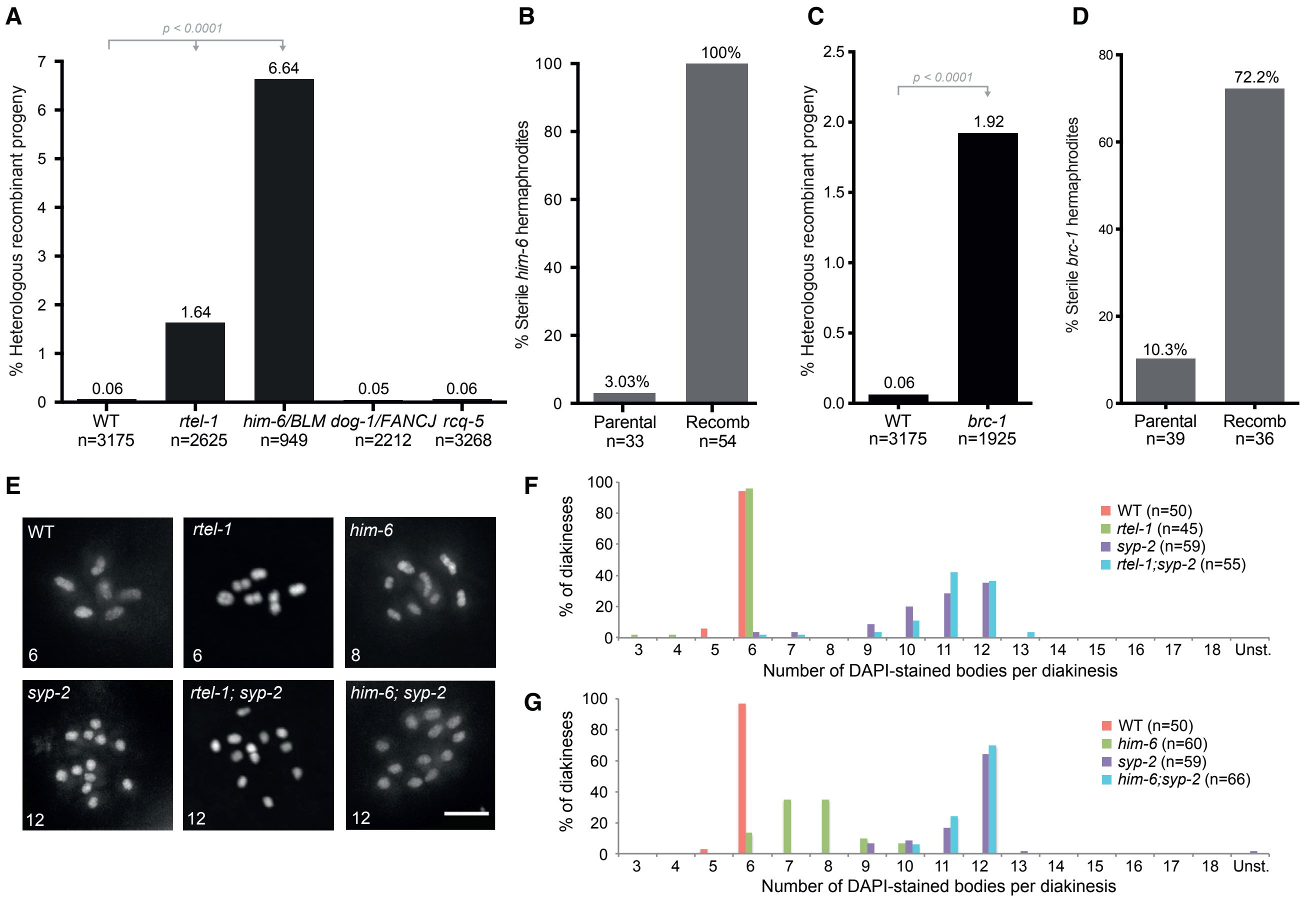

Figure 2. Heterologous Recombination in Helicase Mutants and Inter-sister Repair

(A) Percentage of heterologous recombinant progeny in helicase mutants. n, number of worms scored. Statistics: χ^2 tests.

(B) Percentage of sterile worms among *him-6* parental and *him-6* recombinant hermaphrodites. Statistics: Fisher's exact test.

(C) Percentage of heterologous recombinant progeny in *brc-1* mutants. n, number of worms scored. Statistics: χ^2 tests.

(D) Percentage of sterile among *brc-1* parental and *brc-1* recombinant hermaphrodites. Statistics: Fisher's exact test.

(E) Representative diakineses stained with DAPI. The number of DAPI-stained bodies detectable through the z stack is indicated on each image. Scale bar, 5 μm.

(F and G) Quantification of the number of DAPI-stained bodies per diakinesis in *rtel-1; syp-2* double mutants (F), *him-6; syp-2* double mutants (G), and control genotypes. The number of diakineses scored for each genotype is indicated.

See also Table S1.

synaptonemal complex formation is impaired, and repair via the homologous chromosome is impossible. Instead, *syp-2* mutants repair meiotic DSBs by inter-sister repair and present 12 intact univalent chromosomes at diakinesis (Colaiácovo et al., 2002, 2003; Figure 2E). *brc-1*; *syp-2* double mutants present with chromosomal fragments and more than 12 DAPI-stained bodies that result from unrepaired DSBs because of a lack of inter-sister repair (Adamo et al., 2008). In contrast, combining *syp-2* with either *rtel-1* or *him-6* resulted in a maximum of 12 DAPI-stained bodies per diakinesis (Figures 2E–2G). These results establish that RTEL-1 and HIM-6 are dispensable for inter-sister repair.

Mismatch Repair in Heterologous Recombination

The MMR pathway has been implicated in preventing recombination between homeologous sequences. Surprisingly, none of the MMR mutants in *C. elegans* (*msh-2*, *msh-6*, *mlh-1*, and *pms-2*) resulted in a significant increase in Ht-REC in our system (Figure 3A; Table S1). One explanation might be that MMR in worms acts as a backup pathway to prevent Ht-REC and that its importance is masked by RTEL-1, BRC-1, and/or HIM-6; we therefore scored Ht-REC in the double mutants. Strikingly, mutating *msh-2* in *rtel-1* or *brc-1* worms completely suppressed the high levels of Ht-REC (Figure 3B; Table S1), implying that, instead of preventing Ht-REC, MSH-2 promotes it in the absence of these genes. In contrast, mutation of *msh-2* did not affect Ht-REC in the *him-6* background (Figure 3B; Table S1).

We next addressed whether the pro-Ht-REC activity of MSH-2 is shared with the whole MMR pathway. Mutation of *msh-6*, but not *pms-2*, could also suppress Ht-REC in *rtel-1* (Figure 3C; Table S1), arguing that it is the MSH-2/MSH-6 heterodimer that acts to drive Ht-REC in the absence of RTEL-1 or BRC-1. Intriguingly, mutation of *mlh-1* significantly increases Ht-REC

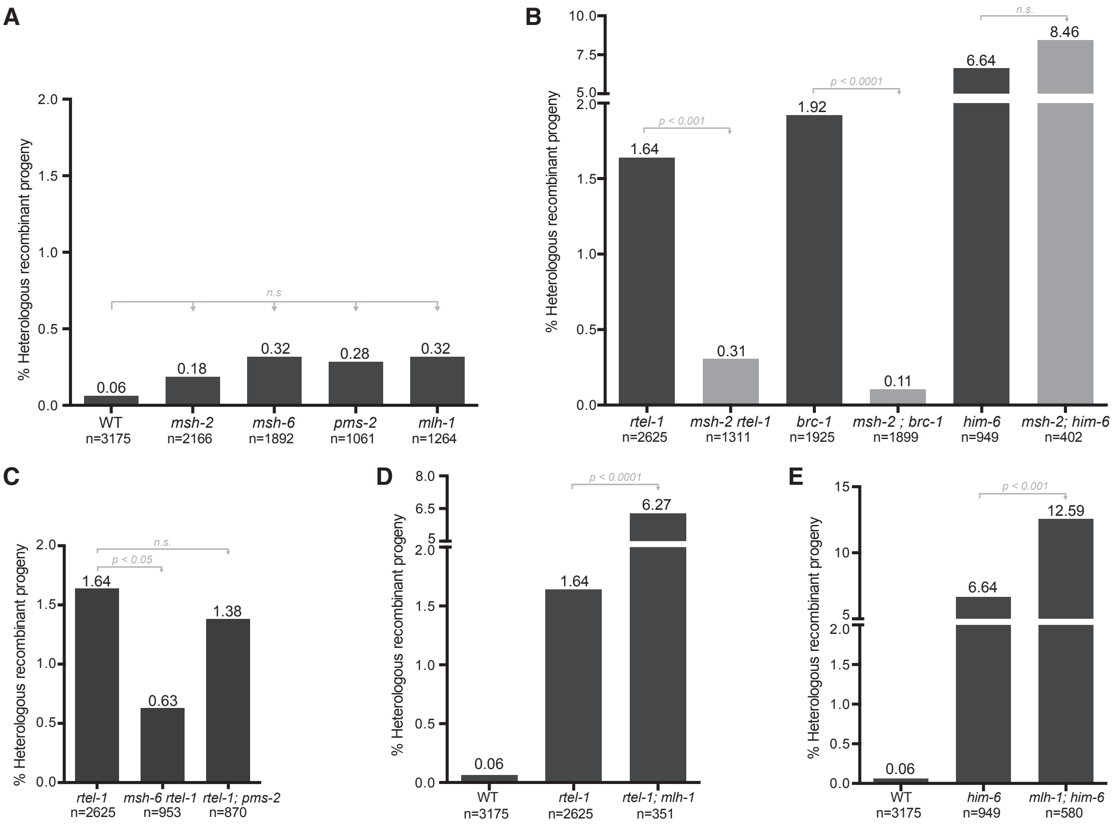

Figure 3. MSH2/MSH6 Are Responsible for the Events in the Absence of *rtel-1* and *brc-1*, but Not in *him-6*

(A–E) Percentage of heterologous recombinant progeny in mismatch repair mutants (A), *msh-2* combined with *rtel-1*, *brc-1* or *him-6* (B), *msh-6* and *pms-2* combined with *rtel-1* (C), *mlh-1* combined with *rtel-1* (D), and *mlh-1* combined with *him-6* (E). n, number of worms scored. Statistics: χ^2 tests or Fisher's exact tests when n $\leq$ 1000.

See also Table S1.

both in *rtel-1* and in *him-6* mutants (Figures 3D and 3E; Table S1), arguing that MLH-1 plays an anti-recombinogenic function in this setting, similar to its previously reported role in preventing homeologous recombination (Nicholson et al., 2000). Finally, these observations reveal a separation of function of the different MMR factors in the control of Ht-REC and demonstrate that the mechanisms that prevent Ht-REC and homeologous recombination are fundamentally different.

MSH-2 Is Responsible for the Additional Crossovers of *rtel-1* Mutants in the Context of Normal Meiosis

Our findings raised the possibility that MSH-2/6 may also cause other *rtel-1* phenotypes (Barber et al., 2008; Youds et al., 2010). Although mutation of *msh-2* failed to suppress the reduction in brood size or the embryonic lethality of *rtel-1 mus-81* or *rtel-1 dog-1* double mutants (Figure 4A; Figure S3A), it partially alleviated the reduction in brood size of the *rtel-1; rcq-5* double mutant (Figure S3A) but not its embryonic lethality (Figure 4A). *msh-2* mutation also partially alleviated the reduction in brood size and the embryonic lethality of *rtel-1; him-6* mutants (Figures S3B and S3C).

We next assessed meiotic crossover formation in *rtel-1* mutants by measuring genetic map distances between visible markers flanking two intervals on chromosomes III and V. Although the map distance for both intervals are comparable between wild-type and *msh-2* mutant worms, indicating that MSH2/6 does not affect the frequency of COs in normal meiosis, loss of MSH-2 rescued the hyper-recombination phenotype of *rtel-1* mutants to wild-type levels (Figure 4B). This result demonstrates that MSH-2 also drives the additional COs that occur during meiosis in the absence of *rtel-1*.

Mouse *Msh2* Is Not Responsible for the Telomere and Replication Phenotypes of *Rtel1* Mutants

We next addressed whether the antagonistic functions for RTEL-1 and MSH-2 found in worms to influence recombination outcomes is relevant in higher eukaryotes. We started by asking whether *Msh2* is responsible for the replication defects and telomere dysfunction previously reported for *Rtel1*-deficient mouse cells (Ding et al., 2004; Vannier et al., 2012, 2013) and generated two clonal *Rtel1*$^{flox/flox}$ mouse embryonic fibroblasts lines in which *Msh2* was knocked out by CRISPR/Cas9 (KO1 and KO2; Figures S3D and S4).

RTEL1 is required to unwind t-loops and DNA G-quadruplex (G4-DNA) structures at telomeres (Vannier et al., 2012). In *Rtel1*-deficient cells, failure to unwind t-loops leads to catastrophic

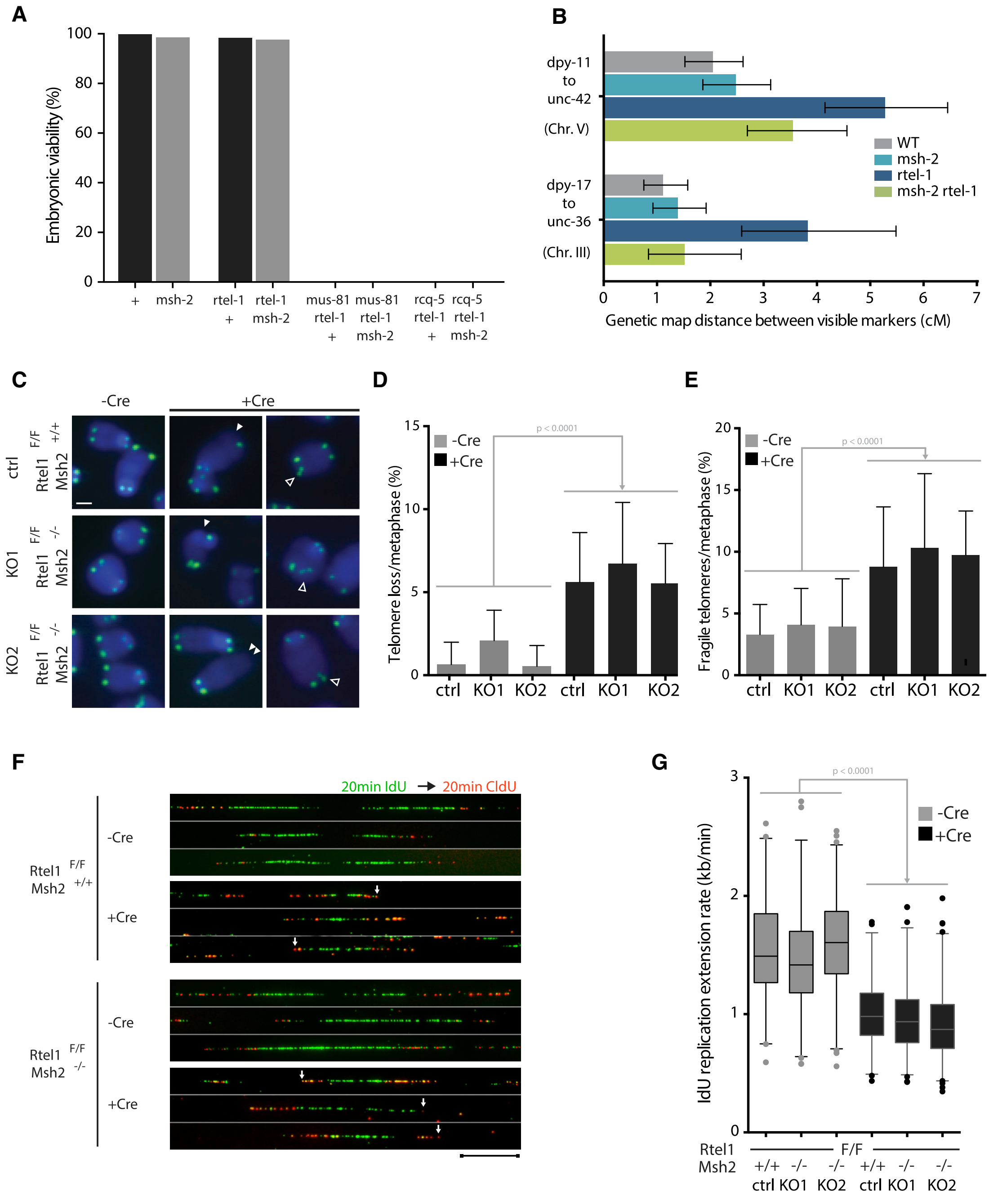

Figure 4. Deletion of *msh-2*/*Msh2* Suppresses Some, but Not All, *rtel-1*/*Rtel1* Phenotypes

(A) Percentage of viable embryos for worms of the indicated genotypes. At least 700 embryos were scored for each genotype.

(B) Recombination frequency as measured by genetic map distance between pairs of marker genes for two chromosomal intervals. Error bars, 95% confidence interval (CI).

(C) Metaphases were analyzed upon excision of *Rtel1* in control or *Msh2* knockout cell lines. Full arrowheads, telomere loss; empty arrowheads, telomere fragility. Telomeric fluorescence *in situ* hybridization (FISH), green; DAPI, blue. Scale bar, 10 μm.

(D and E) Quantification of the number of telomeres lost (D) or the number of fragile telomeres (E) per metaphase from two independent experiments. Statistics: one-way ANOVA. Data represent mean ± SD from at least 50 metaphases.

(F and G) Replication fork dynamics in $Rtel1^{F/F}$ and $Rtel1^{F/F}$, $Msh2^{-/-}$ upon excision of *Rtel1*. The cells were pulse-labeled with iododeoxyuridine (IdU, green) and chlorodeoxyuridine (CldU, red) and subjected to DNA combing (F). The replication origin for each image has been aligned to the center of the image. Scale bar, 12 μm, 24 kb. Arrows, fork stalling or collapse. Replication fork speed was measured in kb min^{-1} for at least one hundred fibers per genotype in two independent experiments (G). Statistics: one-way ANOVA.

See also Figure S4.

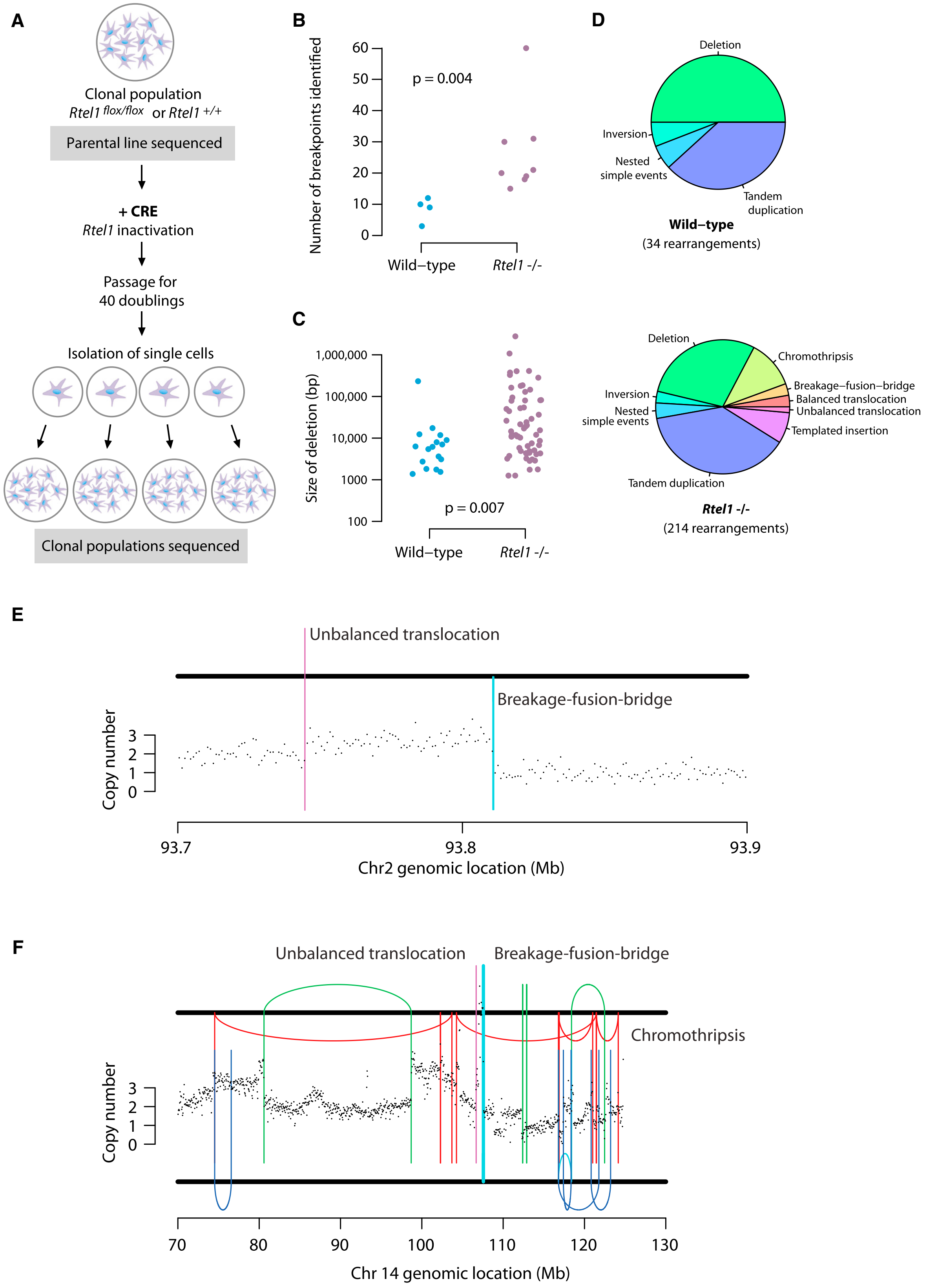

(legend on next page)

processing of the telomere by the SLX1/4 nucleases complex, causing rapid changes in telomere length, increased telomeric sister-chromatid exchanges (T-SCEs), and accumulation of extra-chromosomal t-circles. Conversely, failure to unwind telomeric-G4 DNA structures leads to increased telomere fragility, which is exacerbated by replication inhibition (Vannier et al., 2012). Inactivation of *Rtel1* resulted in an increase in t-circle formation regardless of the status of *Msh2* (Figures S3E and S3F; Vannier et al., 2012). Comparable levels of telomere shortening and loss and telomere fragility were also evident in $Rtel1^{-/-}$ and $Rtel1^{-/-}Msh2^{-/-}$ cells (Figures 4C–4E). Finally, the levels of T-SCEs were similarly increased in *Rtel1*-deficient cells in the presence or absence of *Msh2* (Figures S3G and S3H). Collectively, these results demonstrate that *Msh2* is not responsible for, nor does it contribute to, the telomere phenotypes of *Rtel1*-deficient mouse cells.

During DNA replication, RTEL1 associates with the replisome via its PCNA-interacting PIP box motif to promote replication fork progression. *Rtel1*-deficient cells exhibit increased replication fork stalling and/or collapse, which causes asymmetric progression of sister forks. This, in turn, is thought to trigger increased origin firing, leading to a reduction in inter-origin distances and replication fork extension rates (Vannier et al., 2013). Analysis of replication dynamics revealed that both the symmetry of sister replication forks (Figure 4F) and replication fork extension rates are comparably affected upon inactivation of *Rtel1* in an $Msh2^{+/+}$ or an $Msh2^{-/-}$ background (Figures 4F and 4G), indicating that MSH2 is not the cause of the replication defects that occur in the absence of *Rtel1*.

Loss of *Rtel1* Results in Unusual Classes of Genetic Rearrangements in Mammalian Cells

To address a potential role of RTEL1 in preventing Ht-REC at the genome-wide level in mammalian cells, we characterized structural variations that arise upon conditional loss of *Rtel1* (Figure 5A). Subclones from *Rtel1*-deficient cells showed a higher number of structural variant (SV) breakpoints than those from wild-type cells (Figure 5B; Tables S2 and S3). Wild-type cells had typically 8–12 SVs acquired between rounds of single-cell cloning, whereas *Rtel1*-deficient cells had 15–35 or more breakpoints ($p = 0.004$). In wild-type cells, these structural variants were typically simple intrachromosomal rearrangements, predominantly deletions and tandem duplications, of relatively small size, typically less than 10 kb (Figures 5C and 5D). In contrast, *Rtel1*-deficient cells had both more diversity in the classes of structural variation observed and a wider size distribution for deletions, with many more than 100 kb in size (Figures 5C and 5D).

In keeping with the known actions of RTEL1 at the telomere, we observed inverted "fold-back" rearrangements indicative of end-to-end chromosome fusions, often seen as a feature of breakage-fusion-bridge repair (Figure 5E). In several instances, the breakage-fusion-bridge cycle was followed by a chromothripsis event (Stephens et al., 2011), reminiscent of patterns seen in both *in vitro* models of telomere deficiency (Maciejowski et al., 2015) and human cancers (Li et al., 2014; Figure 5F).

In addition to an increase in relatively simple rearrangements and events associated with telomere dysfunction, *Rtel1*-deficient cells showed a more novel pattern of structural variation (Figure 6). This consisted of duplications of genomic regions that were inserted into the same chromosome at some distance from the original template. The duplicated regions ranged from a few kilobases up to 100 kb in size and were inserted anywhere from 10 kb to several megabases away on the chromosome. At the insertion point, there was typically a small deletion that ranged in size from 6 bp up to 1 kb. Typically, the insertion was in an inverted orientation relative to the original template, although there were insertions that appeared to be non-inverted in orientation. Sometimes, the insertion occurred in the breakpoint of another rearrangement, such as another tandem duplication or a fold-back inversion.

In all instances, these events were associated with an increase in copy number of the original template (Figures 5E and 5F and 6A and 6B). This suggests that this class of structural variation occurs during replication, perhaps through a fork-stalling and template-switching mechanism (Slack et al., 2006). Essentially, a replication fork could stall and switch by Ht-REC to a template kilobases to megabases away and continue replication for tens to hundreds of kilobases in either direction before switching back to within a kilobase or so of the original point of stalling. Such templated duplications with distant intra-chromosomal insertion were not previously observed in *in vitro* models of telomere deficiency (Maciejowski et al., 2015), suggesting that these events are not a consequence of telomere dysfunction.

DISCUSSION

Our study reveals that heterologous recombination is a cause of genome instability and is actively prevented by RTEL-1, HIM-6/BLM, and BRCA1/BRC-1. Although mismatch repair is dispensable for preventing Ht-REC, MSH-2/6 is responsible for driving Ht-REC in *rtel-1* and *brc-1* mutants but not in *him-6/BLM*. We further demonstrate that mutation of *msh-2/Msh2* does not suppress all of the previously described phenotypes of *rtel-1/Rtel1* mutant worms and mouse cells but does abolish the additional

Figure 5. *Rtel1*-Deficient Cells Show a Large Number of Diverse and Complex Structural Variations

(A) Experimental design for deep-sequencing *Rtel1*-deficient cells. Clonal populations of mouse embryonic fibroblasts were isolated from $Rtel1^{+/+}$ and $Rtel1^{flox/flox}$ littermates. *Rtel1* was excised using CRE recombinase, and the resulting *Rtel1*-deficient cells were expanded for 40 doublings. $Rtel1^{+/+}$ and $Rtel1^{-/-}$ subclones were then isolated for deep sequencing.

(B) Number of breakpoints identified in $Rtel1^{+/+}$ and $Rtel1^{-/-}$ subclones. Statistics: Wilcoxon test.

(C) Size distribution of the deletions detected in $Rtel1^{+/+}$ and $Rtel1^{-/-}$ subclones. Statistics: Wilcoxon test.

(D) Pie chart illustrating the proportions of each class of structural variation in $Rtel1^{+/+}$ and $Rtel1^{-/-}$ subclones.

(E) Rearrangement and copy number profile of a breakage-fusion-bridge event on chromosome 2 in $Rtel1^{-/-}$.

(F) Rearrangement and copy number profile of a breakage-fusion-bridge event followed by chromothripsis on chromosome 14 in $Rtel1^{-/-}$.

See also Tables S2 and S3.

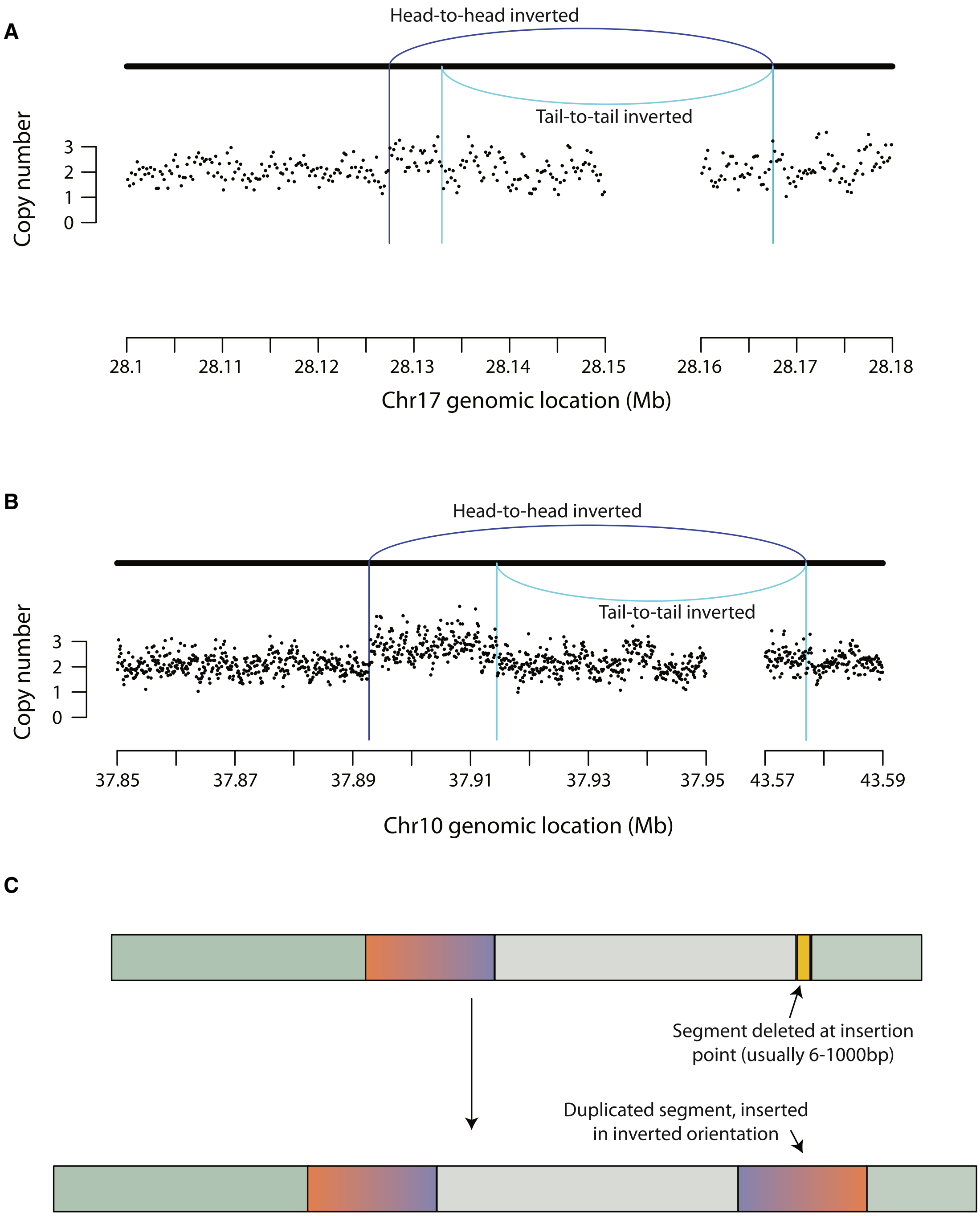

Figure 6. *Rtel1*-Deficient Cells Show a Novel Pattern of Structural Variations

(A and B) Rearrangement and copy number profiles of duplications of regions inserted at some distance and in an inverted orientation on the same chromosome.
(C) Graphical explanation of the events depicted in (A) and (B).

COs that arise during normal meiosis. This finding highlights the physiological relevance of antagonistic functions of RTEL-1 and MSH-2 with respect to Ht-REC. Importantly, we find that deletion of *Rtel1* in mouse cells drives a myriad of unusual structural variations that may arise as a consequence of Ht-REC events occurring during DNA replication. Broadly, our data suggest that Ht-REC is a potential source of genome instability that could explain a number of complex genome rearrangements in cancer genomes.

Heterologous Recombination during Worm Meiosis?

Although there are several possible explanations for our data, we believe that the erroneous repair events observed in

rtel-1, *him-6/BLM*, and *brc-1* mutant worms most likely correspond to *bona fide* Ht-REC events. This is important to clarify, given that an inversion, as used in our study, might be expected to re-orient in a manner that allows its homologous pairing and synapsis to generate classical homologous recombination products. First, although homologous pairing and synapsis have been described previously for inversions in *Drosophila* (Gong et al., 2005), heterologous synapsis is known to occur in different inversion models, including birds and mice (Ashley and Russell, 1986; del Priore and Pigozzi, 2015). Second, chromosome pairing in worms is initiated at well-defined pairing centers, and synapsis propagates independent of sequence homology from these centers along the chromosomes, even between non-homologous chromosomes (MacQueen et al., 2005); after the synaptonemal complex has been assembled, this structure constitutes a robust physical constraint within which a reorientation of the inversion seems highly unlikely. Third, if the recombinant worms were generated by gene conversion as a result of homologous recombination with a reoriented inversion, then they should not display signs of genome instability and sterility. The fact that the vast majority of recombinants are sterile and genetically unstable argues against a gene conversion event following inversion reorientation. Forth, although inter-homolog CO formation increases in the absence of *rtel-1* during normal meiosis (Barber et al., 2008; Youds et al., 2010), COs actually decrease in *him-6* and remain unchanged in *brc-1* mutants (Wicky et al., 2004); that recombination increases in both of these mutants in our inversion system strongly argues for a fundamentally different mechanism distinct from canonical HR. Fifth, the implication of the MSH-2/MSH-6 complex in promoting COs in our assay suggests the presence of mismatches within the intermediates of the recombination products generated, consistent with recombination occurring between heterologous sequences. Finally, mutation of either *lig-4* or *polq-1* did not abolish these events but, instead, elevated erroneous repair in the inversion assay, in complete agreement with previous findings demonstrating that loss of NHEJ or MMEJ enhances recombination (e.g., Pierce et al., 2001; Zelensky et al., 2017). This result excludes beyond any reasonable doubt that NHEJ and MMEJ are promoting the repair events in this system. Collectively, these data strongly argue that the erroneous repair events observed in *rtel-1*, *him-6/BLM*, and *brc-1* worms within the *mIn1* inversion are generated by a recombination-dependent mechanism that is heterologous in nature.

Model for Ht-REC in Worms

C. elegans possesses a single RecA family member (RAD-51) that must perform the role of both Rad51 and Dmc1 in meiosis. Importantly, *C. elegans* RAD-51 can tolerate mismatches during the strand exchange reaction *in vitro* and is biochemically most similar to yeast Dmc1 (O. Belan and E. Greene, personal communication). This intrinsic property of *C. elegans* RAD-51 will permit strand invasion between heterologous sequences containing mismatches, with the resulting recombination intermediates being removed by the action of at least two pathways to prevent Ht-REC (Figure 7A). The first relies on the ability of RTEL-1 to dismantle unstable D-loops that form when strand invasion occurs between heterologous sequences. Following disruption of the D-loop, such DSBs are repaired using the identical sister chromatid, precluding illegitimate CO formation. In *rtel-1* mutants, however, mismatch-containing D-loops are instead recognized and stabilized by the MSH-2/MSH-6 heterodimer, leading to CO formation. As during normal *rtel-1* meiosis, MUS-81 likely processes Ht-REC intermediates in favor of a CO outcome (Osman et al., 2003; Youds et al., 2010). We suggest that it is the inability to repair DSBs through the sister chromatid that is responsible for the increase in Ht-REC in *brc-1* mutants. Given the temporal barrier to inter-sister repair during meiosis (Hayashi et al., 2007), such a defect would be revealed in late pachytene, forcing the breaks to invade the heterologous chromosome and undergo repair to produce illegitimate COs in a manner that is MSH-2/MSH-6- and MUS-81-dependent.

The second pathway depends on the HIM-6/BLM helicase, which likely acts to dissolve dHJs formed at heterologous sequences. In the absence of dissolution in *him-6* mutants, dHJs may be acted upon by structure-specific endonucleases that resolve the dHJ to produce COs. In addition to the increase in sister chromatid exchanges that results from mitotic crossover (Chaganti et al., 1974), BLM-deficient cells display a wide array of chromosomal rearrangements, including deletions, duplications, and translocations (Luo et al., 2000), the origin of which has remained unclear. Rearrangement breakpoints in mouse BLM cells are often located at repetitive sequences and associated with microhomologies at the junction sites (Yamanishi et al., 2013), and genome rearrangements that occur in *Drosophila Blm* mutants arise independently of NHEJ (Garcia et al., 2011), all supporting a role for BLM in promoting NCO repair to prevent Ht-REC. Finally, this function may provide an explanation for the long-observed instability of the ribosomal DNA in yeast *sgs1* mutants and BS cells (Gangloff et al., 1994; Killen et al., 2009).

What Is the Difference between Homeologous and Ht-REC?

That MMR has no effect alone on Ht-REC is perhaps surprising, given its importance in preventing homeologous recombination. This sole observation argues that the mechanisms employed to limit homeologous recombination and Ht-REC are fundamentally different; the difference may lie in the degree of sequence homology between the molecules undergoing recombination. Although sequences used in homeologous recombination studies typically share at least 70% homology (for example, Chambers et al., 1996; Chen and Jinks-Robertson, 1998, 1999; Hunter et al., 1996; Selva et al., 1995), the sequences undergoing recombination in our system are expected to share less homology. Consistent with this idea, studies in yeast suggest that the dependency on MMR to prevent homeologous recombination is lost beyond a certain degree of sequence divergence (Datta et al., 1997; Datta and Jinks-Robertson, 1995; Porter et al., 1996). An anti-recombinogenic function for MLH-1 was, however, revealed in our system upon deletion of *rtel-1* or *him-6/BLM* and may be reminiscent of the role of the yeast Mlh1 protein in homeologous recombination (Nicholson et al., 2000). Nevertheless, our data suggest that MLH-1 acts in a

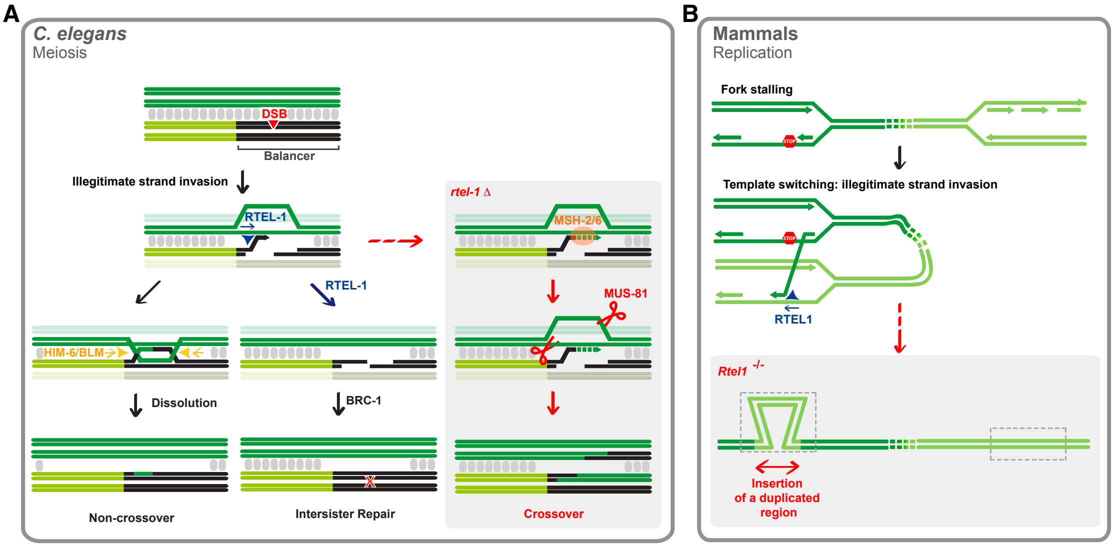

Figure 7. Model: Suppression of Heterologous Recombination in Worms and Mammals

(A) Ht-REC during worm meiosis. Two synapsed homologous chromosomes are represented in light and dark green, one of which contains a region of heterology (black). The synaptonemal complex is represented as gray blocks between the chromosomes. Programmed meiotic DSBs (red triangle) can occur along the chromosomes regardless of the regions of heterology. In wild-type worms, DSBs that occur within this region will attempt strand invasion into the heterologous chromosome and will undergo one of at least two fates. (1) RTEL-1 counteracts strand invasion and D-loop formation, blocking the repair of the DSB through the heterologous chromosome. The break is later repaired through the sister chromatid, which requires the function of BRC-1. In an *rtel-1* mutant, MSH-2/MSH-6 recognizes an intermediate (for example, the heterologous D-loop) and stabilizes it to promote crossover formation through Ht-REC, possibly in an MUS-81-dependent manner. (2) Strand invasion is successful despite the lack of complete homology, and a D-loop is formed. Upon second end capture, a dHJ forms that is dissolved by the HIM-6/BLM helicase and results in a non-crossover event. In a *him-6* mutant, the dHJ is instead resolved by structure-specific endonucleases, which can lead to the formation of crossover events.

(B) Ht-REC during mammalian DNA replication. Two replication forks are represented in a single chromosome. Upon fork stalling, the extending strand changes template, invading a heterologous single-stranded DNA in another replication fork. In a wild-type context, RTEL1 unwinds the resulting heterologous D-loop, thus reverting the template switch. In the absence of *Rtel1*, the D-loop persists and is extended. After the newly synthesized strand is displaced, replication resumes within the initial replication fork and results in duplication of the template sequence that becomes inserted where the initial fork stalled.

backup pathway to RTEL-1 and HIM-6/BLM to prevent Ht-REC in worms.

How Does MSH2/MSH6 Act to Promote Crossing Over?

Mutation of *msh-2/Msh2* abolishes not only Ht-REC in worms but also the additional meiotic crossovers in the absence of *rtel-1*. A similar substrate most likely accumulates and is acted upon by MSH2/6 in both circumstances. First, MSH2/6 may recognize a DNA secondary structure that forms independently of the degree of sequence homology in *rtel-1* mutants. This would be reminiscent of the ability of MutS homologs to bind DNA secondary structures, and, in particular, both the MSH4/MSH5 meiosis-specific heterodimer and the yeast and human MSH2/MSH6 complexes can bind Holliday junctions (Alani et al., 1997; Marsischky et al., 1999; Snowden et al., 2004). Second, MSH-2/6 may act by removing large non-homologous 3′ tails to allow recombination to proceed at internal microhomologies, as does the MSH2/3 complex in *S. cerevisiae* (Pâques and Haber, 1997; Saparbaev et al., 1996; Sugawara et al., 1997). Finally, the recognition by MSH-2/6 may require the presence of mismatches within a recombination intermediate. Consistent with this hypothesis, the human MSH2/6 heterodimer can specifically bind mismatch-containing D-loops *in vitro* (Honda et al., 2014). This third scenario raises the unanticipated possibility that the additional meiotic crossovers of *rtel-1* mutants occur at non-identical sites between the homologous chromosomes (for example, at repetitive DNA sequences), which would provide an explanation for why RTEL-1 counteracts a subset of CO events during worm meiosis while leaving the obligate COs, which might occur at perfectly homologous sequences, unperturbed (Youds et al., 2010). This third scenario may also explain why the telomere and replication phenotypes of *Rtel1* cells are unaffected by the status of *Msh2* because these functions may not involve processing of heterologous sequences during recombination.

RTEL1 Limits a New Class of Genome Rearrangements in Mammals

We find that loss of *Rtel1* also causes the accumulation of a number of structural variations in mouse cells in as little as 40 cell doublings. Among these SVs are chromosome end-to-end fusions, which result from breakage-fusion-bridge cycles and chromothripsis. These are reminiscent of the events observed in *in vitro* models of telomere deficiency (Maciejowski et al., 2015) and can thus be attributed to a failure in maintaining telomere integrity in the absence of RTEL1, as described previously (Vannier et al., 2012). Strikingly, we also find that loss of RTEL1 leads to the formation of very large chromosomal deletions as well as duplications of large regions that are inserted within the same chromosome at variable distances from the original template. Because

the latter involve an increase in copy number, we believe that these events are associated with DNA replication and may initiate at sites of replication fork stalling or collapse, mishaps that commonly occur in the absence of RTEL1 (Vannier et al., 2013). We propose that, upon replication fork stalling, the 3′ prime end attempts strand invasion into a heterologous sequence within the same chromosome. In wild-type cells, this attempt is reversed by the action of RTEL1, which unwinds the heterologous D-loop. In the absence of RTEL1, however, the D-loop persists, and subsequent DNA synthesis occurs, achieving template switching. If the newly synthesized strand is displaced, then a duplication will have occurred that becomes inserted where the initial fork stalled (Figure 7B). Our observations support a role for RTEL1 in dismantling heterologous D-loops and preventing Ht-REC during replication stress in mouse cells.

In an unpublished pan-cancer analysis such rearrangements are also observed within the 2,800 whole-cancer genome sequences (P.J.C., unpublished data). When they are present in human cancers, the duplications with distant intrachromosomal insertion appear to correlate with the frequency of simple deletions of medium and large size, reminiscent of the marked increase in deletions of more than 10 kb seen in the *Rtel1* knockout lines here.

Implications

Overall, these findings define an unappreciated class of genome rearrangements involving heterologous regions of the genome. With the identification of several key players that act to prevent and promote Ht-REC, it should now be possible to examine the relative contribution of MMEJ and Ht-REC to genome instability in cancer through interrogation of cancer genome sequences. With the emergence of RTEL1 mutations in Hoyeraal-Hreidarsson syndrome and variant association with predisposition to a range of brain and other cancers (Sarek et al., 2015; Vannier et al., 2014), it will also be important to examine how signatures of Ht-REC correlate with *RTEL1* status in these diseases and whether these events and the associated phenotypes of affected individuals/mouse models can be alleviated by MSH2/6 inhibition.

STAR★METHODS

Detailed methods are provided in the online version of this paper and include the following:

- KEY RESOURCES TABLE
- CONTACT FOR REAGENT AND RESOURCE SHARING
- EXPERIMENTAL MODEL AND SUBJECT DETAILS
  - Nematode strains
  - Cell lines
- METHOD DETAILS
  - Generation of the mIn1 inversion
  - Cytological preparation of worm germlines
  - Scoring crossover events in *C. elegans*
  - Cre recombination
  - CRISPR
  - Immunoblotting
  - PNA-FISH
  - Chromosome Orientation FISH
  - DNA combing
  - Telomere Circle Assay
  - Copy number and rearrangement analysis
- QUANTIFICATION AND STATISTICAL ANALYSIS

SUPPLEMENTAL INFORMATION

Supplemental Information includes four figures and three tables and can be found with this article online at https://doi.org/10.1016/j.molcel.2017.12.014.

ACKNOWLEDGMENTS

We would like to thank Mark Edgley for different versions of the *mIn1* inversion and J. Stingele, M.R.G. Taylor, and R. Bellelli for comments on the manuscript. We are grateful to members of the S.J.B. laboratory for discussions throughout the project. Work in the S.J.B. lab was supported by the Francis Crick Institute, which receives its core funding from Cancer Research UK (FC0010048), the UK Medical Research Council (FC0010048), and the Wellcome Trust (FC0010048). S.J.B. is also funded by European Research Council (ERC) Advanced Investigator Grants RecMitMei and TelMetab (742437) and a Wellcome Trust senior investigator grant. Work in the P.J.C. lab was supported by the Wellcome Trust (077012/Z/05/Z), and P.J.C. holds a Wellcome Trust senior clinical fellowship (WT088340MA).

AUTHOR CONTRIBUTIONS

A.M.L.-O. and S.J.B. conceived and supervised the study and wrote the paper. A.M.L.-O. performed all of the *C. elegans* Ht-REC analyses, produced mouse cells for sequencing analysis, and quantified all experiments. S.P. generated *Msh2* CRISPR knockouts in mouse cells. S.P. and G.S. conducted the telomere analysis in mouse cells, and J.-B.V. conducted the DNA combing analysis. H.P. advised on bioinformatics, and P.J.C. performed sequencing and bioinformatics on the mouse cells.

DECLARATION OF INTERESTS

The authors declare no competing interests.

Received: July 25, 2017
Revised: October 2, 2017
Accepted: December 18, 2017
Published: January 18, 2018

REFERENCES

Adamo, A., Montemauri, P., Silva, N., Ward, J.D., Boulton, S.J., and La Volpe, A. (2008). BRC-1 acts in the inter-sister pathway of meiotic double-strand break repair. EMBO Rep. *9*, 287–292.

Alani, E., Reenan, R.A., and Kolodner, R.D. (1994). Interaction between mismatch repair and genetic recombination in Saccharomyces cerevisiae. Genetics *137*, 19–39.

Alani, E., Lee, S., Kane, M.F., Griffith, J., and Kolodner, R.D. (1997). Saccharomyces cerevisiae MSH2, a mispaired base recognition protein, also recognizes Holliday junctions in DNA. J. Mol. Biol. *265*, 289–301.

Ashley, T., and Russell, L.B. (1986). A new type of nonhomologous synapsis in T(X;4)1R1 translocation male mice. Cytogenet. Cell Genet. *43*, 194–200.

Barber, L.J., Youds, J.L., Ward, J.D., McIlwraith, M.J., O'Neil, N.J., Petalcorin, M.I., Martin, J.S., Collis, S.J., Cantor, S.B., Auclair, M., et al. (2008). RTEL1 maintains genomic stability by suppressing homologous recombination. Cell *135*, 261–271.

Beadle, G.W., and Sturtevant, A.H. (1935). X Chromosome Inversions and Meiosis in Drosophila Melanogaster. Proc. Natl. Acad. Sci. USA *21*, 384–390.

Brenner, S. (1974). The genetics of Caenorhabditis elegans. Genetics *77*, 71–94.

Bunting, S.F., and Nussenzweig, A. (2013). End-joining, translocations and cancer. Nat. Rev. Cancer *13*, 443–454.

Chaganti, R.S., Schonberg, S., and German, J. (1974). A manyfold increase in sister chromatid exchanges in Bloom's syndrome lymphocytes. Proc. Natl. Acad. Sci. USA *71*, 4508–4512.

Chambers, S.R., Hunter, N., Louis, E.J., and Borts, R.H. (1996). The mismatch repair system reduces meiotic homeologous recombination and stimulates recombination-dependent chromosome loss. Mol. Cell. Biol. *16*, 6110–6120.

Chapman, J.R., Taylor, M.R., and Boulton, S.J. (2012). Playing the end game: DNA double-strand break repair pathway choice. Mol. Cell *47*, 497–510.

Chen, W., and Jinks-Robertson, S. (1998). Mismatch repair proteins regulate heteroduplex formation during mitotic recombination in yeast. Mol. Cell. Biol. *18*, 6525–6537.

Chen, W., and Jinks-Robertson, S. (1999). The role of the mismatch repair machinery in regulating mitotic and meiotic recombination between diverged sequences in yeast. Genetics *151*, 1299–1313.

Colaiácovo, M.P., Stanfield, G.M., Reddy, K.C., Reinke, V., Kim, S.K., and Villeneuve, A.M. (2002). A targeted RNAi screen for genes involved in chromosome morphogenesis and nuclear organization in the Caenorhabditis elegans germline. Genetics *162*, 113–128.

Colaiácovo, M.P., MacQueen, A.J., Martinez-Perez, E., McDonald, K., Adamo, A., La Volpe, A., and Villeneuve, A.M. (2003). Synaptonemal complex assembly in C. elegans is dispensable for loading strand-exchange proteins but critical for proper completion of recombination. Dev. Cell *5*, 463–474.

Daley, J.M., and Sung, P. (2014). 53BP1, BRCA1, and the choice between recombination and end joining at DNA double-strand breaks. Mol. Cell. Biol. *34*, 1380–1388.

Datta, A., and Jinks-Robertson, S. (1995). Association of increased spontaneous mutation rates with high levels of transcription in yeast. Science *268*, 1616–1619.

Datta, A., Adjiri, A., New, L., Crouse, G.F., and Jinks Robertson, S. (1996). Mitotic crossovers between diverged sequences are regulated by mismatch repair proteins in Saccharomyces cerevisiae. Mol. Cell. Biol. *16*, 1085–1093.

Datta, A., Hendrix, M., Lipsitch, M., and Jinks-Robertson, S. (1997). Dual roles for DNA sequence identity and the mismatch repair system in the regulation of mitotic crossing-over in yeast. Proc. Natl. Acad. Sci. USA *94*, 9757–9762.

de Wind, N., Dekker, M., Berns, A., Radman, M., and te Riele, H. (1995). Inactivation of the mouse Msh2 gene results in mismatch repair deficiency, methylation tolerance, hyperrecombination, and predisposition to cancer. Cell *82*, 321–330.

del Priore, L., and Pigozzi, M.I. (2015). Heterologous Synapsis and Crossover Suppression in Heterozygotes for a Pericentric Inversion in the Zebra Finch. Cytogenet. Genome Res. *147*, 154–160.

Detloff, P., White, M.A., and Petes, T.D. (1992). Analysis of a gene conversion gradient at the HIS4 locus in Saccharomyces cerevisiae. Genetics *132*, 113–123.

Ding, H., Schertzer, M., Wu, X., Gertsenstein, M., Selig, S., Kammori, M., Pourvali, R., Poon, S., Vulto, I., Chavez, E., et al. (2004). Regulation of murine telomere length by Rtel: an essential gene encoding a helicase-like protein. Cell *117*, 873–886.

Edgley, M.L., and Riddle, D.L. (2001). LG II balancer chromosomes in Caenorhabditis elegans: mT1(II;III) and the mIn1 set of dominantly and recessively marked inversions. Mol. Genet. Genomics *266*, 385–395.

Gangloff, S., McDonald, J.P., Bendixen, C., Arthur, L., and Rothstein, R. (1994). The yeast type I topoisomerase Top3 interacts with Sgs1, a DNA helicase homolog: a potential eukaryotic reverse gyrase. Mol. Cell. Biol. *14*, 8391–8398.

Garcia, A.M., Salomon, R.N., Witsell, A., Liepkalns, J., Calder, R.B., Lee, M., Lundell, M., Vijg, J., and McVey, M. (2011). Loss of the bloom syndrome helicase increases DNA ligase 4-independent genome rearrangements and tumorigenesis in aging Drosophila. Genome Biol. *12*, R121.

Gong, W.J., McKim, K.S., and Hawley, R.S. (2005). All paired up with no place to go: pairing, synapsis, and DSB formation in a balancer heterozygote. PLoS Genet. *1*, e67.

Hayashi, M., Chin, G.M., and Villeneuve, A.M. (2007). C. elegans germ cells switch between distinct modes of double-strand break repair during meiotic prophase progression. PLoS Genet. *3*, e191.

Honda, M., Okuno, Y., Hengel, S.R., Martín-López, J.V., Cook, C.P., Amunugama, R., Soukup, R.J., Subramanyam, S., Fishel, R., and Spies, M. (2014). Mismatch repair protein hMSH2-hMSH6 recognizes mismatches and forms sliding clamps within a D-loop recombination intermediate. Proc. Natl. Acad. Sci. USA *111*, E316–E325.

Hu, Y., Raynard, S., Sehorn, M.G., Lu, X., Bussen, W., Zheng, L., Stark, J.M., Barnes, E.L., Chi, P., Janscak, P., et al. (2007). RECQL5/Recql5 helicase regulates homologous recombination and suppresses tumor formation via disruption of Rad51 presynaptic filaments. Genes Dev. *21*, 3073–3084.

Hunter, N., Chambers, S.R., Louis, E.J., and Borts, R.H. (1996). The mismatch repair system contributes to meiotic sterility in an interspecific yeast hybrid. EMBO J. *15*, 1726–1733.

Jasin, M., and Rothstein, R. (2013). Repair of strand breaks by homologous recombination. Cold Spring Harb. Perspect. Biol. *5*, a012740.

Killen, M.W., Stults, D.M., Adachi, N., Hanakahi, L., and Pierce, A.J. (2009). Loss of Bloom syndrome protein destabilizes human gene cluster architecture. Hum. Mol. Genet. *18*, 3417–3428.

Lansdorp, P.M., Verwoerd, N.P., van de Rijke, F.M., Dragowska, V., Little, M.T., Dirks, R.W., Raap, A.K., and Tanke, H.J. (1996). Heterogeneity in telomere length of human chromosomes. Hum. Mol. Genet. *5*, 685–691.

Larocque, J.R., and Jasin, M. (2010). Mechanisms of recombination between diverged sequences in wild-type and BLM-deficient mouse and human cells. Mol. Cell. Biol. *30*, 1887–1897.

Li, Y., Schwab, C., Ryan, S., Papaemmanuil, E., Robinson, H.M., Jacobs, P., Moorman, A.V., Dyer, S., Borrow, J., Griffiths, M., et al. (2014). Constitutional and somatic rearrangement of chromosome 21 in acute lymphoblastic leukaemia. Nature *508*, 98–102.

Luo, G., Santoro, I.M., McDaniel, L.D., Nishijima, I., Mills, M., Youssoufian, H., Vogel, H., Schultz, R.A., and Bradley, A. (2000). Cancer predisposition caused by elevated mitotic recombination in Bloom mice. Nat. Genet. *26*, 424–429.

Maciejowski, J., Li, Y., Bosco, N., Campbell, P.J., and de Lange, T. (2015). Chromothripsis and Kataegis Induced by Telomere Crisis. Cell *163*, 1641–1654.

MacQueen, A.J., Phillips, C.M., Bhalla, N., Weiser, P., Villeneuve, A.M., and Dernburg, A.F. (2005). Chromosome sites play dual roles to establish homologous synapsis during meiosis in C. elegans. Cell *123*, 1037–1050.

Mankouri, H.W., and Hickson, I.D. (2007). The RecQ helicase-topoisomerase III-Rmi1 complex: a DNA structure-specific 'dissolvasome'? Trends Biochem. Sci. *32*, 538–546.

Marsischky, G.T., Lee, S., Griffith, J., and Kolodner, R.D. (1999). 'Saccharomyces cerevisiae MSH2/6 complex interacts with Holliday junctions and facilitates their cleavage by phage resolution enzymes. J. Biol. Chem. *274*, 7200–7206.

Myung, K., Datta, A., Chen, C., and Kolodner, R.D. (2001). SGS1, the Saccharomyces cerevisiae homologue of BLM and WRN, suppresses genome instability and homeologous recombination. Nat. Genet. *27*, 113–116.

Nicholson, A., Hendrix, M., Jinks-Robertson, S., and Crouse, G.F. (2000). Regulation of mitotic homeologous recombination in yeast. Functions of mismatch repair and nucleotide excision repair genes. Genetics *154*, 133–146.

Novitski, E., and Braver, G. (1954). An Analysis of Crossing over within a Heterozygous Inversion in Drosophila Melanogaster. Genetics *39*, 197–209.

Osman, F., Dixon, J., Doe, C.L., and Whitby, M.C. (2003). Generating crossovers by resolution of nicked Holliday junctions: a role for Mus81-Eme1 in meiosis. Mol. Cell *12*, 761–774.

Pâques, F., and Haber, J.E. (1997). Two pathways for removal of nonhomologous DNA ends during double-strand break repair in Saccharomyces cerevisiae. Mol. Cell. Biol. *17*, 6765–6771.

Pierce, A.J., Hu, P., Han, M., Ellis, N., and Jasin, M. (2001). Ku DNA end-binding protein modulates homologous repair of double-strand breaks in mammalian cells. Genes Dev. *15*, 3237–3242.

Porter, G., Westmoreland, J., Priebe, S., and Resnick, M.A. (1996). Homologous and homeologous intermolecular gene conversion are not differentially affected by mutations in the DNA damage or the mismatch repair genes RAD1, RAD50, RAD51, RAD52, RAD54, PMS1 and MSH2. Genetics *143*, 755–767.

Sanjana, N.E., Shalem, O., and Zhang, F. (2014). Improved vectors and genome-wide libraries for CRISPR screening. Nat. Methods *11*, 783–784.

Saparbaev, M., Prakash, L., and Prakash, S. (1996). Requirement of mismatch repair genes MSH2 and MSH3 in the RAD1-RAD10 pathway of mitotic recombination in Saccharomyces cerevisiae. Genetics *142*, 727–736.

Sarek, G., Marzec, P., Margalef, P., and Boulton, S.J. (2015). Molecular basis of telomere dysfunction in human genetic diseases. Nat. Struct. Mol. Biol. *22*, 867–874.

Selva, E.M., New, L., Crouse, G.F., and Lahue, R.S. (1995). Mismatch correction acts as a barrier to homeologous recombination in Saccharomyces cerevisiae. Genetics *139*, 1175–1188.

Sfeir, A., and Symington, L.S. (2015). Microhomology-Mediated End Joining: A Back-up Survival Mechanism or Dedicated Pathway? Trends Biochem. Sci. *40*, 701–714.

Shalem, O., Sanjana, N.E., Hartenian, E., Shi, X., Scott, D.A., Mikkelson, T., Heckl, D., Ebert, B.L., Root, D.E., Doench, J.G., and Zhang, F. (2014). Genome-scale CRISPR-Cas9 knockout screening in human cells. Science *343*, 84–87.

Slack, A., Thornton, P.C., Magner, D.B., Rosenberg, S.M., and Hastings, P.J. (2006). On the mechanism of gene amplification induced under stress in Escherichia coli. PLoS Genet. *2*, e48.

Snowden, T., Acharya, S., Butz, C., Berardini, M., and Fishel, R. (2004). hMSH4-hMSH5 recognizes Holliday Junctions and forms a meiosis-specific sliding clamp that embraces homologous chromosomes. Mol. Cell *15*, 437–451.

Spell, R.M., and Jinks-Robertson, S. (2004). Examination of the roles of Sgs1 and Srs2 helicases in the enforcement of recombination fidelity in Saccharomyces cerevisiae. Genetics *168*, 1855–1865.

Stephens, P.J., Greenman, C.D., Fu, B., Yang, F., Bignell, G.R., Mudie, L.J., Pleasance, E.D., Lau, K.W., Beare, D., Stebbings, L.A., et al. (2011). Massive genomic rearrangement acquired in a single catastrophic event during cancer development. Cell *144*, 27–40.

Sugawara, N., Pâques, F., Colaiácovo, M., and Haber, J.E. (1997). Role of Saccharomyces cerevisiae Msh2 and Msh3 repair proteins in double-strand break-induced recombination. Proc. Natl. Acad. Sci. USA *94*, 9214–9219.

Tay, Y.D., Sidebotham, J.M., and Wu, L. (2010). Mph1 requires mismatch repair-independent and -dependent functions of MutSalpha to regulate crossover formation during homologous recombination repair. Nucleic Acids Res. *38*, 1889–1901.

Tham, K.C., Hermans, N., Winterwerp, H.H., Cox, M.M., Wyman, C., Kanaar, R., and Lebbink, J.H. (2013). Mismatch repair inhibits homeologous recombination via coordinated directional unwinding of trapped DNA structures. Mol. Cell *51*, 326–337.

Truong, L.N., Li, Y., Shi, L.Z., Hwang, P.Y., He, J., Wang, H., Razavian, N., Berns, M.W., and Wu, X. (2013). Microhomology-mediated End Joining and Homologous Recombination share the initial end resection step to repair DNA double-strand breaks in mammalian cells. Proc. Natl. Acad. Sci. USA *110*, 7720–7725.

Vannier, J.B., Pavicic-Kaltenbrunner, V., Petalcorin, M.I., Ding, H., and Boulton, S.J. (2012). RTEL1 dismantles T loops and counteracts telomeric G4-DNA to maintain telomere integrity. Cell *149*, 795–806.

Vannier, J.B., Sandhu, S., Petalcorin, M.I., Wu, X., Nabi, Z., Ding, H., and Boulton, S.J. (2013). RTEL1 is a replisome-associated helicase that promotes telomere and genome-wide replication. Science *342*, 239–242.

Vannier, J.B., Sarek, G., and Boulton, S.J. (2014). RTEL1: functions of a disease-associated helicase. Trends Cell Biol. *24*, 416–425.

Welz-Voegele, C., and Jinks-Robertson, S. (2008). Sequence divergence impedes crossover more than noncrossover events during mitotic gap repair in yeast. Genetics *179*, 1251–1262.

West, S.C., Blanco, M.G., Chan, Y.W., Matos, J., Sarbajna, S., and Wyatt, H.D. (2015). Resolution of recombination intermediates: mechanisms and regulation. Cold Spring Harb. Symp. Quant. Biol. *80*, 103–109.

Westmoreland, J., Porter, G., Radman, M., and Resnick, M.A. (1997). Highly mismatched molecules resembling recombination intermediates efficiently transform mismatch repair proficient Escherichia coli. Genetics *145*, 29–38.

Wicky, C., Alpi, A., Passannante, M., Rose, A., Gartner, A., and Müller, F. (2004). Multiple genetic pathways involving the Caenorhabditis elegans Bloom's syndrome genes him-6, rad-51, and top-3 are needed to maintain genome stability in the germ line. Mol. Cell. Biol. *24*, 5016–5027.

Worth, L., Jr., Clark, S., Radman, M., and Modrich, P. (1994). Mismatch repair proteins MutS and MutL inhibit RecA-catalyzed strand transfer between diverged DNAs. Proc. Natl. Acad. Sci. USA *91*, 3238–3241.

Worth, L., Jr., Bader, T., Yang, J., and Clark, S. (1998). Role of MutS ATPase activity in MutS,L-dependent block of in vitro strand transfer. J. Biol. Chem. *273*, 23176–23182.

Wu, L., and Hickson, I.D. (2003). The Bloom's syndrome helicase suppresses crossing over during homologous recombination. Nature *426*, 870–874.

Xu, D., Guo, R., Sobeck, A., Bachrati, C.Z., Yang, J., Enomoto, T., Brown, G.W., Hoatlin, M.E., Hickson, I.D., and Wang, W. (2008). RMI, a new OB-fold complex essential for Bloom syndrome protein to maintain genome stability. Genes Dev. *22*, 2843–2855.

Yamanishi, A., Yusa, K., Horie, K., Tokunaga, M., Kusano, K., Kokubu, C., and Takeda, J. (2013). Enhancement of microhomology-mediated genomic rearrangements by transient loss of mouse Bloom syndrome helicase. Genome Res. *23*, 1462–1473.

Youds, J.L., Mets, D.G., McIlwraith, M.J., Martin, J.S., Ward, J.D., ONeil, N.J., Rose, A.M., West, S.C., Meyer, B.J., and Boulton, S.J. (2010). RTEL-1 enforces meiotic crossover interference and homeostasis. Science *327*, 1254–1258.

Zelensky, A.N., Schimmel, J., Kool, H., Kanaar, R., and Tijsterman, M. (2017). Inactivation of Pol θ and C-NHEJ eliminates off-target integration of exogenous DNA. Nat. Commun. *8*, 66.

Zetka, M.C., and Rose, A.M. (1992). The meiotic behavior of an inversion in Caenorhabditis elegans. Genetics *131*, 321–332.

STAR★METHODS

KEY RESOURCES TABLE

REAGENT or RESOURCE	SOURCE	IDENTIFIER
Antibodies		
Mouse Monoclonal anti-FLAG(R) M2	Sigma-Aldrich	Cat# F1804; RRID: AB_262044
Rabbit Monoclonal anti-MSH2	Cell Signaling	Cat# 2017S; RRID: AB_2235387
Mouse Monoclonal anti-alpha-Tubulin	Sigma-Aldrich	Cat#T6074; RRID: AB_477582
Rat Polyclonal anti-BrdU	Santa Cruz Biotechnology	Cat#sc-56258; RRID: AB_781696
Mouse Anti-BrdU Monoclonal Antibody, FITC Conjugated, Clone B44	BD Biosciences	Cat#347583; RRID: AB_400327
Peroxidase-conjugated Goat anti-Mouse IgG (H+L)	Thermo Fisher Scientific	Cat#G-21040; RRID: AB_2536527
Peroxidase-conjugated Goat anti-Rabbit IgG (H+L)	Thermo Fisher Scientific	Cat#G-21234; RRID: AB_2536530
Chicken Anti-Goat IgG (H+L) Antibody, Alexa Fluor594 Conjugated	Thermo Fisher	Cat#A-21468; RRID: AB_141859
Donkey Anti-Rabbit IgG (H+L) Antibody, Alexa Fluor488 Conjugated	Thermo Fisher	Cat#A-21206; RRID: AB_141708
Goat Anti-Rat IgG (H+L) Antibody, Alexa Fluor594 Conjugated	Thermo Fisher	Cat#A-11007; RRID: AB_141374
Rabbit Anti-Mouse IgG (H+L) Antibody, Alexa Fluor488 Conjugated	Thermo Fisher	Cat#A-11059; RRID: AB_142495
Chemicals, Peptides, and Recombinant Proteins		
Adenovirus Ad-Cre-GFP	Vector Biolabs	Cat#1700
Adenovirus Ad-GFP	Vector Biolabs	Cat#1060
Blocking Reagent	Sigma-Aldrich	Cat#11096176001 ROCHE
BrdU	Sigma-Aldrich	Cat#B9285
CldU	Sigma-Aldrich	Cat#C6891
Colcemid	Sigma-Aldrich	Cat#0295892001 ROCHE
EDTA-free Complete protease inhibitor cocktail	Roche	Cat#COEDTAF-RO
Exonuclease III	Promega	Cat#M1815
Hoechst 33258	Sigma-Aldrich	Cat#861405
IdU	Sigma-Aldrich	Cat#I7125
Levamisole	Sigma-Aldrich	Cat#196142
Phi29 DNA Polymerase	Thermo Fisher	Cat#EP0091
PhosSTOP phosphatase inhibitor cocktail	Roche	Cat#PHOSS-RO
Poly-L-Lysine	Sigma-Aldrich	Cat#4707
Critical Commercial Assays		
FiberPrep (DNA Extraction Kit)	Genomic Vision	Cat#EXTR-001
ProLong Gold antifade with DAPI	Thermo Fisher	Cat#P36931
Vectashield	Vectorlabs	Cat#H-1200
Virapower	Thermo Fisher	Cat#K497500
Lipofectamine 2000	Thermo Fisher	Cat#12566014
Deposited Data		
Raw Sequence data (ENA PRJEB10906)	This study	https://www.ebi.ac.uk/ena/data/view/PRJEB10906
Experimental Models: Cell Lines		
Mouse Embryonic Fibroblasts *Rtel1*$^{f/f}$	Vannier et al., 2012	N/A
Mouse Embryonic Fibroblasts *Rtel1*$^{f/f}$*Msh2*$^{-/-}$	This study	N/A
293FT	Thermo Fisher	Cat#R70007

(*Continued on next page*)

Continued

REAGENT or RESOURCE	SOURCE	IDENTIFIER
Mouse Ear Fibroblasts *Rtel1*$^{+/+}$	This study	N/A
Mouse Ear Fibroblasts *Rtel1*$^{f/f}$	This study	N/A
Experimental Models: Organisms/Strains		
C. elegans: WT, Bristol (N2) background.	CGC	N2
C. elegans: *mIn1[mIs14 rol-1(e91)]/dpy-25(e817) II.*	This study	DW579
C. elegans: *rtel-1(tm1866) I; mIn1[mIs14 rol-1(e91)]/dpy-25(e817) II.*	This study	DW581
C. elegans: *lig-4(ok716) III; mIn1[mIs14 rol-1(e91)]/dpy-25(e817) II.*	This study	DW599
C. elegans: *mIn1[mIs14 rol-1(e91)]/dpy-25(e817) II; polq-1(tm2026)/hT2 III.*	This study	DW755
C. elegans: *him-6(ok412) IV; mIn1[mIs14 rol-1(e91)]/dpy-25(e817) II.*	This study	DW627
C. elegans: *rcq-5(tm424) III ; mIn1[mIs14 rol-1(e91)]/dpy-25(e817) II.*	This study	DW594
C. elegans: *dog-1(gk10) I; mIn1[mIs14 rol-1(e91)]/dpy-25(e817) II.*	This study	DW588
C. elegans: *brc-1(tm1145) III; mIn1[mIs14 rol-1(e91)]/dpy-25(e817) II.*	This study	DW624
C. elegans: *msh-2(ok2410) I; mIn1[mIs14 rol-1(e91)]/dpy-25(e817) II.*	This study	DW623
C. elegans: *msh-6(pk2504) I; mIn1[mIs14 rol-1(e91)]/dpy-25(e817) II.*	This study	DW580
C. elegans: *mlh-1(ok1917) III; mIn1[mIs14 rol-1(e91)]/dpy-25(e817) II.*	This study	DW696
C. elegans: *unc-4(e120) rol-1(e91)/mIn1[dpy-10(e128) mIs14] II.*	This study	DW549
C. elegans: *rtel-1(tm1866); unc-4(e120) rol-1(e91)/mIn1 [dpy-10(e128) mIs14] II.*	This study	DW552
C. elegans: *rtel-1(tm1866) I; lig-4(ok716) III; mIn1[mIs14 rol-1(e91)]/dpy-25(e817) II.*	This study	DW609:
C. elegans: *rtel-1(tm1866)I; mIn1[mIs14 rol-1(e91)]/dpy-25(e817) II; polq-1(tm2026)/hT2 III.*	This study	DW771
C. elegans: *rtel-1(tm1866) msh-2(ok2410) I; mIn1[mIs14 rol-1(e91)]/dpy-25(e817) II.*	This study	DW628
C. elegans: *msh-2(ok2410) I; mIn1[mIs14 rol-1(e91)]/dpy-25(e817) II; him-6(ok412) IV.*	This study	DW637
C. elegans: *msh-2(ok2410) I; mIn1[mIs14 rol-1(e91)]/dpy-25(e817) II; brc-1(tm1145) III.*	This study	DW648
C. elegans: *msh-6(pk2504) rtel-1(tm1866) I; mIn1[mIs14 rol-1(e91)]/dpy-25(e817) II.*	This study	DW661
C. elegans: *rtel-1(tm1866) I; pms-2(ok2529) V; mIn1[mIs14 rol-1(e91)]/dpy-25(e817) II.*	This study	DW721
C. elegans:: *rtel-1(tm1866) I; mlh-1(ok1917) III; mIn1[mIs14 rol-1(e91)]/dpy-25(e817) II.*	This study	DW741
C. elegans: *mIn1[mIs14 rol-1(e91)]/dpy-25(e817) II;mlh-1(ok1917) III; him-6(ok412) IV.*	This study	DW729
C. elegans: *syp-2 (ok307)V/nT1 [Unc-? (n754) let-? qIs50] (IV;V)*	Laboratory of Monica Colaiacovo	AV276
C. elegans: *rtel-1(tm1866)/hT2[gfp] I.*	This study	DW618
C. elegans: *rtel-1(tm1866) I; syp-2 (ok307)V/nT1 [Unc (n754) let-? qIs50] (IV;V)*	This study	DW570

(*Continued on next page*)

Continued

REAGENT or RESOURCE	SOURCE	IDENTIFIER
C. elegans: him-6(ok412) IV	CGC	VC193
C. elegans: him-6(ok412) IV	Outcrossed: this study	DW619
C. elegans: *him-6(ok412)/nT1 IV; syp-2 (ok307)/nT1 [Unc (n754) let-? qls50] V*	This study	DW646
C. elegans: *msh-2(ok2410) rtel-1(tm1866)/hT2[gfp] I.*	This study	DW625
C. elegans: *msh-2(ok2410) mus-81(tm1937) rtel-1 (tm1866) I / hT2[gfp] (I;III).*	This study	DW642
C. elegans: *msh-2(ok2410) rtel-1(tm1866)/hT2 I; rcq-5(tm424) + / + hT2 III.*	This study	DW645
C. elegans: *msh-2(ok2410) rtel-1(tm1866) dog-1 (gk10)/hT2 dog-1(gk10) I; +/hT2 III.*	This study	DW665
C. elegans: *msh-2(ok2410) rtel-1(tm1866) I / hT2[gfp] (I;III); him-6(ok412) IV.*	This study	DW643
C. elegans: *dpy-11(e224) unc-42(e270) V.*	Laboratory of Ann Rose	KR3499
C. elegans: *msh-2(ok2410) I; dpy-11(e224) unc-42(e270) V.*	This study	DW705
C. elegans: *rtel-1(tm1866) I/hT2 (I;III); dpy-11(e224) unc-42(e270) V.*	This study	DW718
C. elegans: *msh-2(ok2410) rtel-1(tm1866) I/hT2 (I;III); dpy-11(e224) unc-42(e270) V.*	This study	DW719
C. elegans: *dpy-17(e164) unc-36(e251) III.*	Laboratory of Ann Rose	KR180
C. elegans: *msh-2(ok2410) I; dpy-17(e164) unc-36(e251) III.*	This study	DW707
C. elegans: *rtel-1(tm1866) I; dpy-17(e164) unc-36(e251) III.*	Barber et al., 2008	DW663
C. elegans: *msh-2(ok2410) rtel-1(tm1866) I; dpy-17(e164) unc-36(e251) III.*	This study	DW664
Oligonucleotides		
FITC-TelC PNA probe	PNA Bio-synthesis	Cat#F1009
TAMRA-TelG PNA probe	PNA Bio-synthesis	Cat#F1006
Thio-TelC oligo: 5′-CCCTAACCCTAACCCTAAccc-3′	Sigma	N/A
TelG oligo: 5′-TTAGGGTTAGGGTTAGGGTTAGGG-3′	Sigma	N/A
Recombinant DNA		
LentiCRISPRv2	Addgene	Cat#52961
Software and Algorithms		
Adobe Photoshop CC	Adobe	http://www.adobe.com/es/products/photoshop.html
GraphPad Prism 7	GraphPad	https://www.graphpad.com/
ImageJ	NIH	https://imagej.nih.gov/ij/
Volocity 6.3	PerkinElmer	http://cellularimaging.perkinelmer.com/downloads/detail.php?id=14
SoftWorx	Applied Precision	https://www.gelifesciences.com/webapp/wcs/stores/servlet/catalog/en/GELifeSciences-uk/brands/deltavision/
GIMP	GIMP	https://www.gimp.org

CONTACT FOR REAGENT AND RESOURCE SHARING

Further information and requests for reagents should be directed to and will be fulfilled by the Lead Contact, Simon Boulton (simon.boulton@crick.ac.uk).

EXPERIMENTAL MODEL AND SUBJECT DETAILS

Nematode strains

All sources of the nematode strains used in the study are listed in the reagent and resource table. Nematode strains were maintained as previously described (Brenner, 1974).

Cell lines

Sources of cell lines used in the study are listed in the reagent and resource table. SV40-LT-immortalized *Rtel1*$^{f/f}$ mouse embryonic fibroblasts and SV40-LT-immortalized *Rtel1*$^{f/f}$ *and Rtel1*$^{+/+}$ *mouse ear fibroblasts* were cultured at 37°C/ 5% CO_2/ 5% O_2 in Dulbecco's modified Eagle's medium (Life Technologies) supplemented with 10% fetal bovine serum (Invitrogen). The sex of the cells was not determined for this study.

METHOD DETAILS

Generation of the mIn1 inversion

The *rol-1(e91)* and the *mIs14(GFP)* markers were linked on the *mIn1* balancer by recombination between *mIn1[rol-1(e91)]* and *mIn1[mIs14(GFP)]*, which were kindly provided by Mark Edgley.

Cytological preparation of worm germlines

Gravid hermaphrodites were transferred to 30 μL PBS on a poly-L-lysine (Sigma-Aldrich) coated slide (slides were washed in 70% ethanol, then given 2 coats of 100% poly-L-lysine, air drying between each coat). The worms were washed in PBS before transferring to 50 μL 10 mM levamisole (Sigma-Aldrich). Germlines were extruded by removing the head and tail using a fine gauge needle (27 G). Levamisole was replaced with 1% paraformaldehyde (Sigma-Aldrich) in PBS for 10 min and germlines were permeabilized for 5 min in TBSBT (TBS+0.5% BSA+0.1% Triton X-100), then washed in TBSB for at least 2 × 5 min and mounted with a coverslip on Vectashield containing DAPI (Vector Laboratories). Deltavision microscopy was used to examine germlines with × 40 or × 63, 1.4 NA Planapochromat lens, and images captured using the SoftWorx computer software (Applied Precision). Three-dimensional datasets were computationally deconvolved, and regions of interest then projected into one dimension. Images were recorded using GIMP software.

Scoring crossover events in *C. elegans*

To score illegitimate recombination between heterologous sequences in *C. elegans*, we made use of the *mIn1* inversion on chromosome II, a genetic balancer that has been previously characterized (Edgley and Riddle, 2001). Genetic balancers are genome rearrangements, typically duplications, reciprocal translocations or inversions that are refractory to COs during meiotic recombination, as they represent locally a region of non- homology with respect to the homologous chromosome. We started with a heterozygous parental worm that carries one normal copy of chromosome II and one copy with the *mIn1* inversion (Figure S1A). The normal copy carries the *dpy-25(e817)* mutation, which is a semi-dominant mutation that causes the worms to be dumpy, i.e., short and fat. The *mIn1* inversion carries the *rol-1(e91)* mutation and an insertion of a GFP-expressing transgene – *mIs14*. The *rol-1* mutation is recessive and causes worms to roll around in circles as they move, while the *mIs14* trangene insertion is semi-dominant and leads to GFP expression from 4-cell embryos to adults, in which it is expressed in the pharynx (Figure S1A). In the absence of recombination across the inversion, a heterozygous *dpy-25/mIn1[rol-1 GFP] II* hermaphrodite is expected to produce the following Mendelian distribution of progeny: 50% of heterozygous *dpy-25/mIn1,* 25% of homozygous *dpy-25/dpy-25* and 25% of homozygous *mIn1/mIn1*, all of which can be distinguished phenotypically (Figures S1B and S1C). If illegitimate CO events occur between *mIn1* and the normal chromosome II, we would expect to see new combinations of phenotypes as depicted in Figures S1B and S1D.

In order to confirm the increase in heterologous recombination observed in the absence of *rtel-1*, we used a different scoring system on the *mIn1* inversion. In this system, a heterozygous parent carried one normal chromosome II marked with the *unc-4(e120)* and the *rol-1(e91)* recessive mutations, which respectively produce uncoordinated and roller worms when homozygous. The other chromosome II carried the *mIn1* inversion marked with the recessive *dpy-10(e128)* mutation, of which homozygous carriers are dumpy. In the absence of recombination, these heterozygous parents should generate the following progeny in mendelian ratios: 25% *unc-4 rol-1 II* (phenotype: [Unc, Rol]), 25% *mIn(dpy-10) II* (phenotype: [Dpy]) and 50% of *unc-4 rol-1/mIn1(dpy-10) II* (phenotype: [non-Unc, non-Rol, non-Dpy]) (Figure S1C). New combinations of phenotypes arise from crossover events between the *mIn1* inversion and the normal chromosome II.

Cre recombination

Rtel1$^{F/F}$ and *Rtel1*$^{F/F}$ *Msh2*$^{-/-}$ cell lines were infected with an adenovirus expressing the Cre recombinase and the GFP marker to inactivate *Rtel1* (Ad-Cre-GFP) or a control adenovirus expressing only GFP (Ad-GFP). A second round of infection was performed after 48 hours, and samples were processed for analysis 96 hours after the first infection. Cells were genotyped by PCR at 96 hr post-infection to confirm gene deletion.

CRISPR

Msh2 was knocked out by CRISPR/Cas9 in immortalized *Rtel1*-conditional mouse embryonic fibroblasts ($Rtel1^{F/F}$) as described in (Sanjana et al., 2014; Shalem et al., 2014). Briefly, we co-expressed Flag-Cas9 and an *Msh2* sgRNA in $Rtel1^{F/F}$ cells using the lentiCRISPRv2 vector system (Sanjana et al., 2014). The sgRNA was designed with the CRISPR Design Tool from Genome Engineering (http://tools.genome-engineering.org) and targets the following sequence in mouse *Msh2*: CAGTTGGAAGGCGCGGCCG. It was cloned into lentiCRISPRv2 (Sanjana et al., 2014) and then, together with ViraPower viral packaging plasmids (Invitrogen) transfected into 293FT cells using Lipofectamine 2000 (Invitrogen) according to the manufacturer's protocol. Lentiviral supernatants were collected 72 h after transfection, filtered through a 0.45-μm filter, and used for spin transduction of $Rtel1^{F/F}$ cells as described above. Transduced cells were subjected for selection 72 h after transduction with 1 μg/ml puromycin. After lentiviral infection, single cell clones were isolated, propagated and analyzed by immunoblotting and sequencing. Two Msh2 knockout clones were selected based on their low levels of Cas9 expression. Exon 1 of the Msh2 gene in these two clones was PCR amplified and Sanger sequenced to determine the nature of the generated indels (Figure S4).

Immunoblotting

Cells were pelleted, washed once with PBS, resuspended in ice-cold lysis buffer (50mM HEPES-KOH (pH 8.0), 100 mM KCl, 2mM EDTA, 0.5% NP-40, 10% sucrose, 1x EDTA-free Complete protease inhibitor cocktail (Roche), 1x PhosSTOP phosphatase inhibitor cocktail (Roche)) and incubated on ice for 20 minutes. The lysates were then passed through a 23G syringe five times. The soluble protein fractions were collected after centrifugation at 16000 x g for 20 minutes at 4°C. Protein lysates were analyzed by immunoblotting using standard SDS–polyacrylamide gel electrophoresis (SDS-PAGE) techniques. In brief, protein samples were boiled in 2X NuPage Sample Buffer (Invitrogen), separated in 4%–12% gradient polyacrylamide gels (Invitrogen), and transferred onto polyvinylidene difluoride membranes (Merck). The membranes were blocked in 1X TBST/ 5% milk for 30 min at room temperature and then incubated with primary antibodies in 1X TBST/ 5% milk overnight at 4°C. The membranes were washed thoroughly with 1X TBST and then incubated with secondary horseradish peroxidase–linked antibodies in 1X TBST/ 5% milk for 1 hour at room temperature. The membranes were again washed thoroughly with 1X TBST and then visualized using the enhanced chemiluminescence reagent from Amersham.

PNA-FISH

Telomeric Peptide Nucleic Acid Fluorescence *In Situ* Hybridization (PNA FISH) was performed as described previously (Lansdorp et al., 1996). Briefly, cells were treated with 0.2 μg/ml of colcemid (Roche) for 90 minutes to arrest cells in metaphase. Trypsinized cells were incubated in 75 mM KCl and fixed with methanol:acetic acid (3:1 ratio). The cells were then dropped onto glass slides and left to dry for at least 24 hours. The slides were rehydrated in PBS for 5 minutes, fixed in 4% formaldehyde for 5 minutes, treated with 1 mg/ml of pepsin for 10 minutes at 37°C, and fixed again in 4% formaldehyde for 5 minutes. Next, slides were dehydrated in 70%, 85%, and 100% (v/v) ethanol for 15 minutes each and then air-dried. Metaphase chromosome spreads were hybridized with a telomeric FITC-TelC PNA probe in hybridization buffer (70% formamide, 0.5% blocking reagent (Roche), 10 mM Tris-HCl pH 7.2) for 1 hour at room temperature, followed by 2 washes in wash buffer (70% formamide, 10mM Tris-HCl pH 7.2) for 15 min each and 3 washes in 1X PBS for 5 min each. The slides were mounted using ProLong Gold antifade with DAPI (Thermo Fisher). Chromosome images and telomere signals were captured using Zeiss Axio Imager M1 microscope equipped with an ORCA-ER camera (Hamamatsu) controlled by Volocity 6.3 software (Improvision).

Chromosome Orientation FISH

Telomeric sister chromatid exchanges were visualized by Chromosome Orientation Fluorescence *In Situ* Hybridization (FISH). Cells were incubated with 10 μM BrdU (Sigma-Aldrich) for 20 hours. Cells were then treated with 0.2 μg/ml of colcemid (Roche) for 90 minutes to arrest cells in metaphase. Trypsinized cells were incubated in 75 mM KCl and fixed with methanol:acetic acid (3:1 ratio). The cells were then dropped onto glass slides and left to dry for at least 24 hours. The slides were rehydrated in PBS for 5 minutes, treated with 0.5 mg/ml RNase A (in 1X PBS) for 15 min at 37°C, stained with 0.5 μg/ml Hoechst 33258 (in 2X SSC) for 20 min at room temperature and finally irradiated at 365 nm UV light (Stratalinker 1800 UV, $5.4 \times 10^3 J/m^2$) for 45 min. The BrdU-labeled DNA strands were digested with 100 μl Exonuclease III (Promega) at 10 U/ μl, in the buffer supplied by the manufacturer, for 30 min at room temperature. The slides were washed in 1X PBS for 5 min, dehydrated in 70%, 85%, and 100% (v/v) ethanol for 5 minutes each and then air-dried. Metaphase chromosome spreads were hybridized with a telomeric FITC-TelC PNA probe in hybridization buffer (70% formamide, 0.5% blocking reagent (Roche), 10 mM Tris-HCl pH 7.2) for 2 hours at room temperature and rinsed in wash buffer 1 (70% formamide, 10mM Tris-HCl pH 7.2, 0.1% BSA). The metaphase chromosome spreads were then hybridized with a telomeric TAMRA-TelG PNA probe in hybridization buffer (70% formamide, 0.5% blocking reagent (Roche), 10 mM Tris-HCl pH 7.2) for 2 hours at room temperature. The slides were washed twice with wash buffer 1 for 15 min each, followed by 3 washes in 1X PBS for 5 min each. Next, the slides were dehydrated in 70%, 85%, and 100% (v/v) ethanol for 5 minutes each, air-dried and mounted using ProLong Gold antifade with DAPI (Thermo Fisher). Chromosome images and telomere signals were captured using Zeiss Axio Imager M1 microscope equipped with an ORCA-ER camera (Hamamatsu) controlled by Volocity 6.3 software (Improvision).

DNA combing

DNA combing was performed essentially as described in (Vannier et al., 2013). Briefly, *Rtel1*$^{f/f}$*Msh2*$^{+/+}$ and *Rtel1*$^{f/f}$*Msh2*$^{-/}$ MEFs were infected with control- or Cre-expressing adenovirus. Cells were pulse-labeled with IdU and CldU for 20 minutes each. Cells were then collected and the DNA was extracted according to DNA extraction kit provided by Genomic Vision. DNA fibers were extracted in agarose plugs and stretched on silanized coverslips with the molecular combing system (Genomic Vision). CldU was detected with rat anti-BrdU antibody (BU1/75, AbCys), followed by goat anti-rat coupled to Alexa 594 (A11007, Molecular Probes) and finally by chicken anti-goat coupled to Alexa 594 (A21468, Molecular Probes). IdU was detected with Mouse anti-BrdU coupled to FITC antibody (BD44, Becton Dickinson), followed by rabbit anti-mouse coupled to Alexa 488 (A11059, Molecular Probes) and finally by donkey anti-rabbit coupled to Alexa 488 (A21206, Molecular Probes). DNA fibers were captured with a Zeiss Axio Imager M1 microscope equipped with an ORCA-ER camera (Hamamatsu) controlled by Volocity 6.3 software (Improvision).

Telomere Circle Assay

Cells grown at a confluence between 70% to 80% were collected from one 10 cm dish. To isolate genomic DNA, cells were resuspended in TNE (10 mM Tris pH 7.4, 10 mM EDTA, 100 mM NaCl) and lysed in TNES (10 mM Tris pH 7.4, 100 mM NaCl, 10 mM EDTA, 1% SDS) in the presence of 100 μg/ml proteinase K (Roche) overnight at 37°C. After phenol/chloroform extraction, the DNA was precipitated with isopropanol, resuspended in TNE + 10mg/ml RNase A (Roche) and incubated for 2 hours at 37°C The DNA was then incubated in TNES + 100 μg/ml proteinase K for 1 hour 37°C followed by a second round of phenol/chloroform extraction and isopropanol precipitation. Pure genomic DNA was resuspend in TE buffer (10 mM Tris pH 8.0, 10 mM EDTA). 3 μg of genomic DNA was digested with AluI/HinfI overnight at 37°C and then ethanol precipitated. The digested DNA was resuspended in 10 μL of 1X annealing buffer (20mM Tris [pH 7.5], 20mM KCl, and 0.1 mM EDTA, 1 μM Thio-TelC primer). The mix was denatured at 96°C for 5 min and cooled down to 25°C for 1 hour. Next, 10 μL of TCA reaction mix (1X Phi29 buffer (Fermentas), 2mM dNTPs, 10 U Phi29 polymerase (Fermentas)) were added to the annealed DNA and incubated at 30°C for 12 hours. Rolling circle amplification was stopped by incubation at 65°C for 20 min. The extension products were separated on a denaturing agarose gel (0.8% agarose, 50 mM NaOH, and 1 mM EDTA pH 8.0) at 3.5 V/cm for 18 hours and transferred onto a positively charged nylon membrane (GE Healthcare) by Southern blotting. Telomere circles were visualized by hybridizing the UV-crosslinked membrane with a γ[32P]-labeled TelG probe. Southern blot images were captured with a Storm 840 scanner. Telomere circle levels were quantified in ImageJ and normalized to control reactions lacking Phi29 polymerase.

Copy number and rearrangement analysis

Ear fibroblasts were harvested from two sets of *Rtel1*$^{f/f}$ *and Rtel1*$^{+/+}$ littermates and immortalized with SV40 T antigen. After Cre treatment, the cells were grown for 40 doublings. Single cell clones were then isolated, amplified and subjected to whole genome sequencing to 30x average coverage. For each *Rtel1*$^{f/f}$ / *Rtel1*$^{+/+}$ set, the parental lines prior to Cre recombinase, as well as four subclones of the *Rtel1*-deficient cells and two subclones of the *Rtel1* wild-type cells were subjected to sequence analysis (Figure 5A). Genomic DNA sequencing libraries were synthesized on robots and cluster generation and sequencing were performed using the manufacturer pipelines. Average sequencing coverage across the samples was 30x. Copy number analysis, rearrangement calling, chromothripsis and other complex structural variations were identified using in-house algorithm's as previously described (Maciejowski et al., 2015).

Raw sequence data for this study: ENA accession number - PRJEB10906.

The link to the sequence data: https://www.ebi.ac.uk/ena/data/view/PRJEB10906

QUANTIFICATION AND STATISTICAL ANALYSIS

Statistical parameters, including statistical tests used, number of events quantified, standard deviation, and statistical significance are reported in the figures and in the figure legends. Statistical analysis has been performed using GraphPad Prism7 software (GraphPad) and statistical significance is determined by the value of $p < 0.05$.

Molecular Cell, Volume *69*

Supplemental Information

A Distinct Class of Genome Rearrangements

Driven by Heterologous Recombination

Ana María León-Ortiz, Stephanie Panier, Grzegorz Sarek, Jean-Baptiste Vannier, Harshil Patel, Peter J. Campbell, and Simon J. Boulton

SUPPLEMENTAL DATA

A Distinct Class of Genome Rearrangements Driven by Heterologous Recombination

Ana María León-Ortiz[1], Stephanie Panier[1], Grzegorz Sarek[1], Jean-Baptiste Vannier[2], Harshil Patel[3], Peter J. Campbell[4] & Simon J. Boulton[1,*]

Supplemental Figure S1. Visual method to score Ht-REC in *Caenorhabditis elegans.*

Supplemental Figure S2. Scoring Ht-REC using a second set of genetic markers.

Supplemental Figure S3. Effect of mutating msh-2/Msh2 on rtel-1/Rtel1 mutant phenotypes.

Supplemental Figure S4. Generating Msh2 CRISPR/Cas9 knockout cell lines.

Supplemental Table S1. Scoring heterologous recombination.

Supplemental Table S2. SVs in Rtel1-deficient subclones (*separate excel file*).

Supplemental Table S3. SVs in wild-type subclones (*separate excel file*).

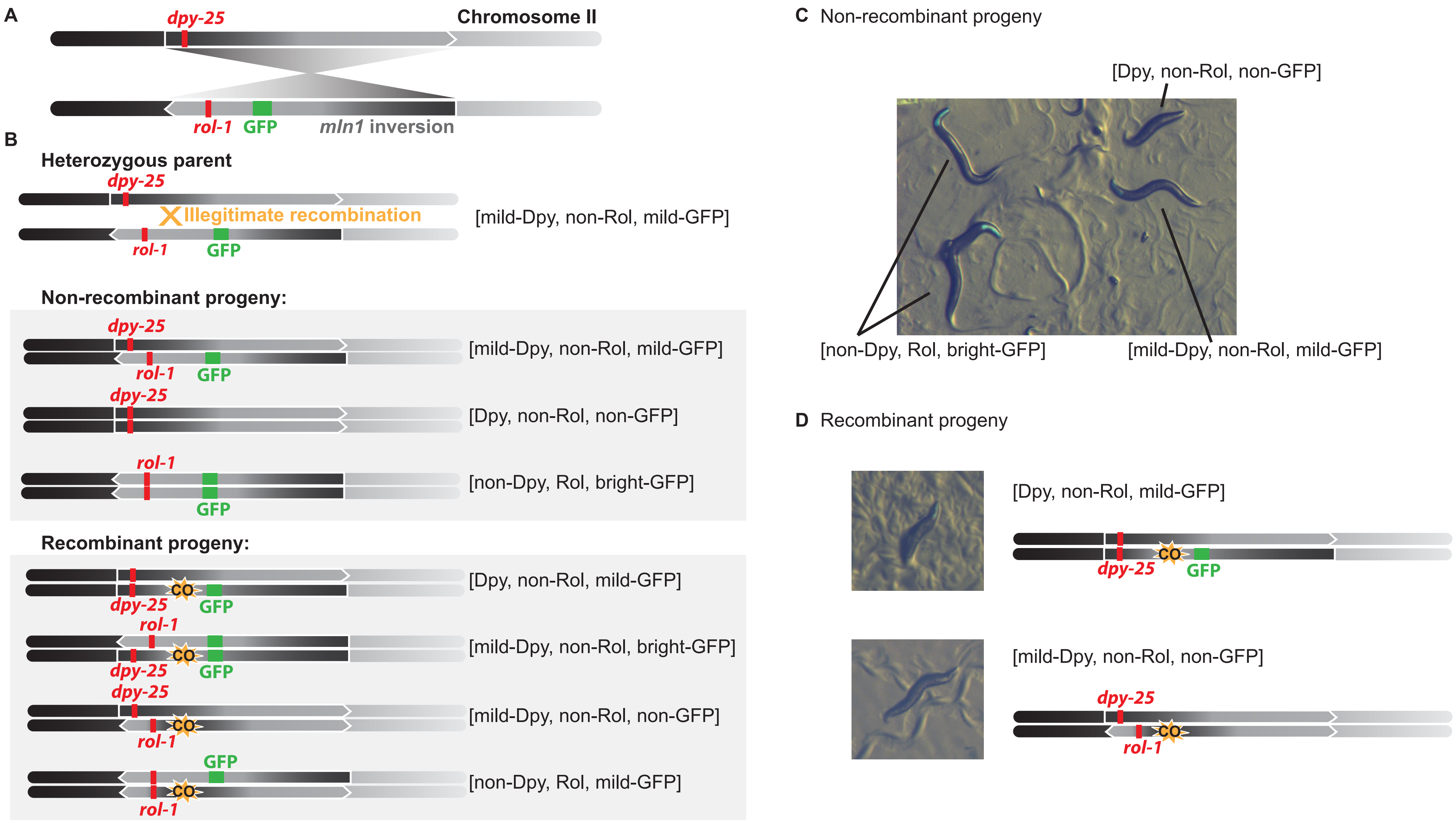

Supplemental Figure S1. Visual method to score Ht-REC in *Caenorbabditis elegans*. Related to Figures 1, 2 and 3.

(A) The system used to score heterologous recombination in *C. elegans* relies on the *mIn1* inversion on chromosome II and visible genetic marker. One copy of chromosome II (top) is marked with the semi-dominant *dpy-25* mutation. The second copy of chromosome II (bottom) contains the *mIn1* inversion marked with the recessive *rol-1* mutation, as well as a semi-dominant insertion of a GFP-expressing transgene.

(B) Recombination is scored in a heterozygous parent that carries one normal copy of chromosome II and one copy that has the *t* inversion. Chromosome organisation and the corresponding phenotypes expected are presented for non-recombinant and for recombinant progeny. CO: crossover location resulting from illegitimate recombination. Note that not all possible recombinant progeny is depicted.

(C-D) Representative images of non-recombinant (C) and recombinant (D) worms. The corresponding phenotypes are indicated for each worm and the diagrams represent a possible crossover event that may explain the phenotypes observed in these worms.

A Heterozygous parent

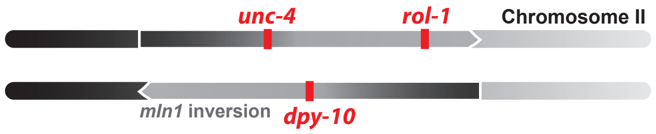

[non-Unc, non-Rol, non-Dpy]

B

	Non-recombinant			Recombinant				
	non-Unc non-Rol non-Dpy	Unc Rol non-Dpy	non-Unc non-Rol Dpy	Unc non-Rol Dpy	non-Unc Rol non-Dpy	non-Unc Rol Dpy	non-Unc Rol non-Dpy	Recombinant / Total (Percent)
WT	2253	826	826	0	0	2	0	2 / 3907 (0.05)
rtel-1	1927	701	980	3	0	46	3	52 / 3660 (1.4)

Supplemental Figure S2. Scoring Ht-REC using a second set of genetic markers. Related to STAR Methods.

(A) Diagram representing the second system used to score heterologous recombination. A heterozygous parent carries one normal chromosome II, marked with recessive *unc-4* and *rol-1* mutations, and one chromosome II with the *mIn1* inversion marked with a recessive *dpy-10* mutation. These parental worms are [non-Unc, non-Rol, non-Dpy].

(B) Table showing the non-recombinant and recombinant progeny scored using the system described in (A) in wild-type and *rtel-1* mutant backgrounds.

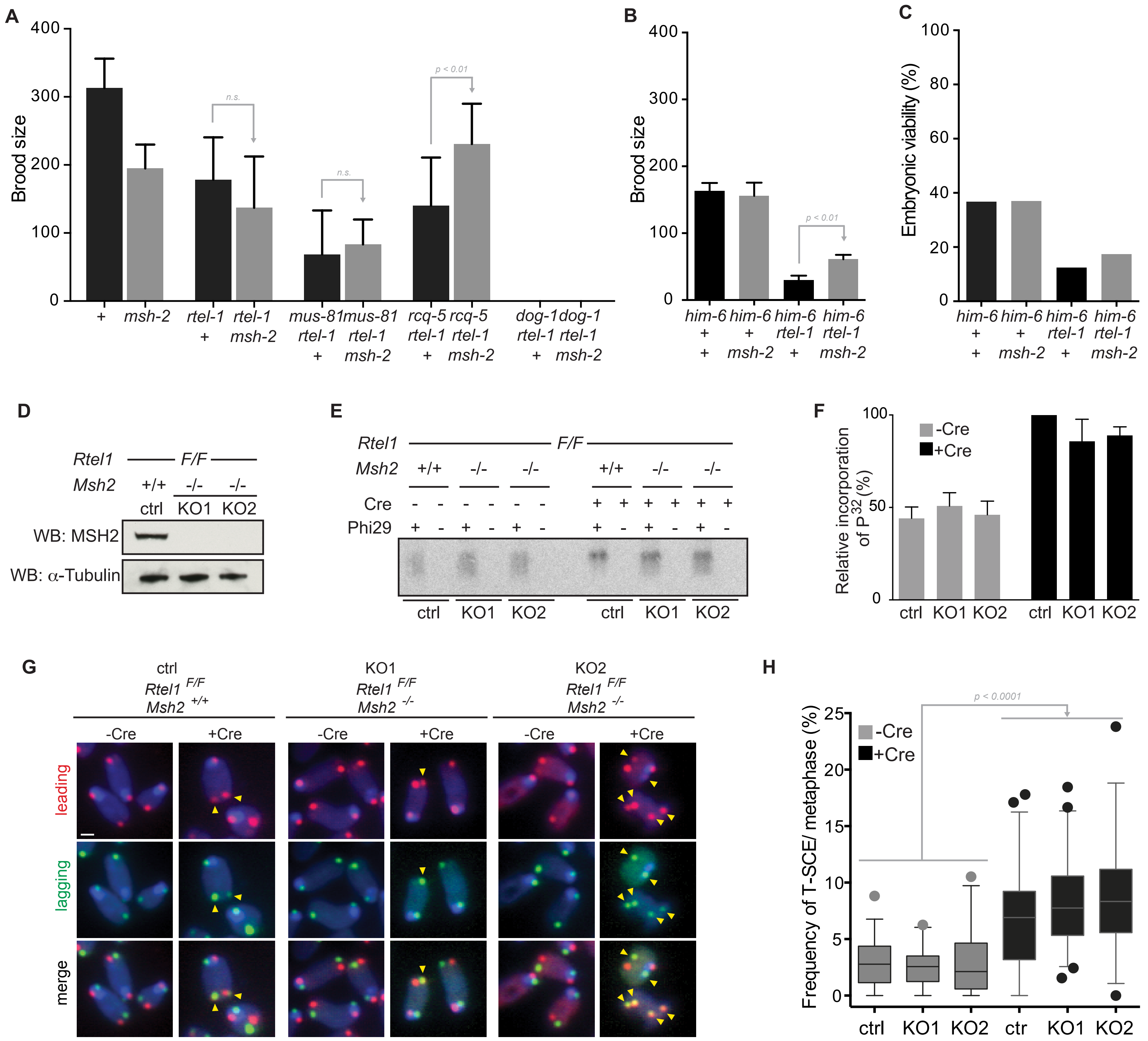
A
B
C
Brood size
Embryonic viability (%)
n.s.
p < 0.01
+ msh-2 rtel-1 + rtel-1 msh-2 mus-81 rtel-1 + mus-81 rtel-1 msh-2 rcq-5 rtel-1 + rcq-5 rtel-1 msh-2 dog-1 rtel-1 + dog-1 rtel-1 msh-2
him-6 + + him-6 + msh-2 him-6 rtel-1 + him-6 rtel-1 msh-2
D
Rtel1 F/F
Msh2 +/+ -/- -/-
ctrl KO1 KO2
WB: MSH2
WB: α-Tubulin
E
Rtel1 F/F
Msh2 +/+ -/- -/- +/+ -/- -/-
Cre
Phi29
ctrl KO1 KO2 ctrl KO1 KO2
F
Relative incorporation of P32 (%)
-Cre
+Cre
ctrl KO1 KO2 ctrl KO1 KO2
G
ctrl Rtel1 F/F Msh2 +/+
KO1 Rtel1 F/F Msh2 -/-
KO2 Rtel1 F/F Msh2 -/-
-Cre +Cre
leading
lagging
merge
H
p < 0.0001
Frequency of T-SCE/ metaphase (%)
-Cre
+Cre
ctrl KO1 KO2 ctr KO1 KO2

Supplemental Figure S3. Effect of mutating *msh-2*/*Msh2* on *rtel-1*/*Rtel1* mutant phenotypes. Related to Figure 4.

(A-B) Brood sizes of the indicated genotypes. The brood size of at least 12 worms was scored for each genotype. Error bars correspond to standard error of the mean. Statistics: Mann-Whitney Wilcoxon test.

(C) Percentage of viable embryos for worms of the indicated genotypes. For each genotype, at least 700 embryos were scored for viability.

(D) Western blot showing loss of Msh2 expression in the CRISPR/Cas9 knockout cell lines KO1 and KO2.

(E-F) T-circle amplification assay upon Cre-driven excision of *Rtel1* in the control or the *Msh2* knockout cell lines (KO1 and KO2). (E) shows a representative image of the assay. Three independent experiments were quantified in (F).

(G-H) Telomere sister-chromatid exchanges visualized by chromosome-oriented FISH on metaphase spreads. Two telomeric probes specific to the leading (green) or the lagging (red) strand were used. Yellow arrowheads indicate overlap between the green and the red signal where recombination between telomeres has taken place (G). Scale bar: 10µm. Two independent experiements were quantified in (H). The graph represents a box and whiskers plot where the whiskers correspond to the 5th-95th

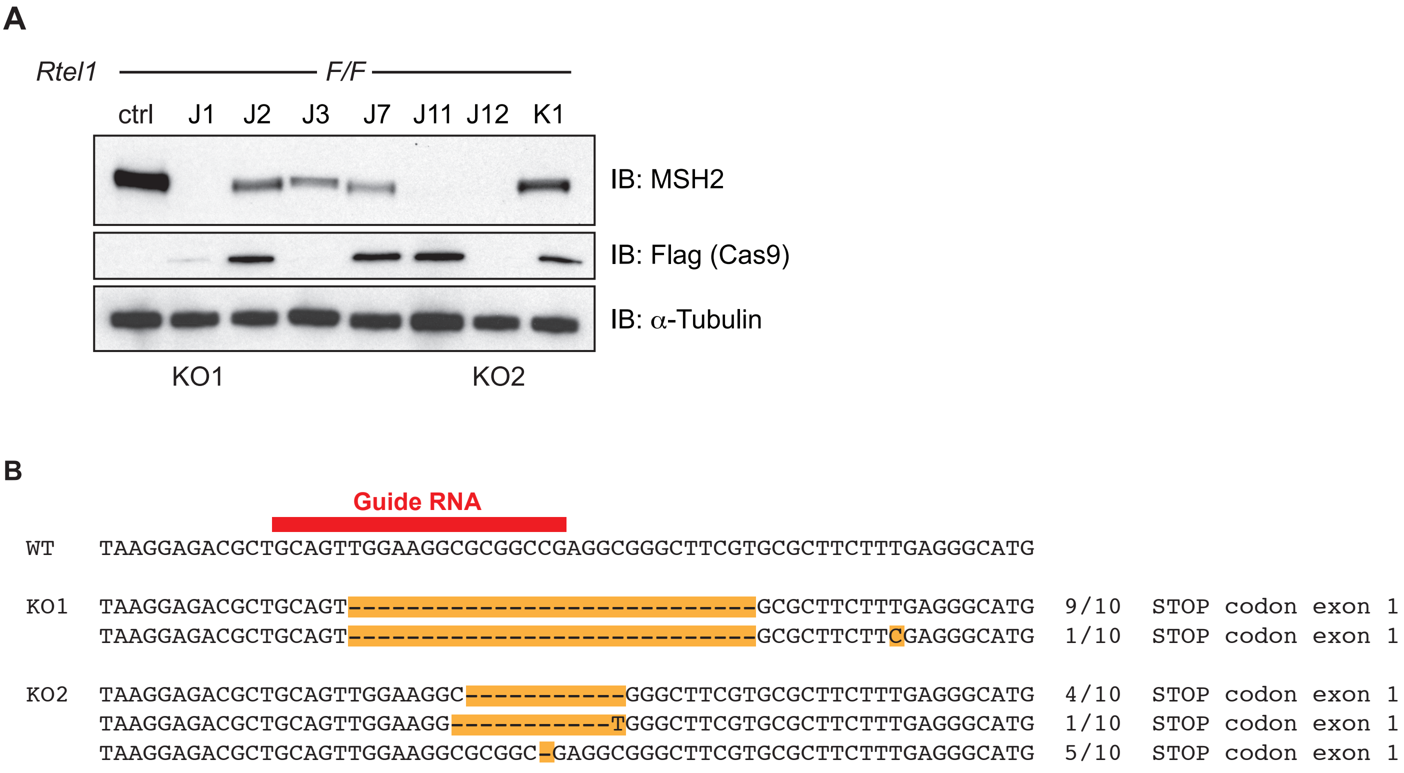

Supplemental Figure S4. Generating Msh2 CRISPR/Cas9 knockout cell lines. Related to Figure S3D and STAR Methods.

(A) Western-blot showing the screening of clonal populations for complete loss of the MSH2 protein. The two knockout cell lines used in this study (labelled KO1 and KO2 under the blots) were chosen on the basis of the low or absent expression of Cas9 (revealed by a Flag antibody).

(B) DNA sequences of the two *Msh2* knockout cell lines used in this study. The sequence targeted by the guide RNA used is shown.

Genotype	Non-recombinant progeny (percent)			Recombinant progeny (percent)					Recombinants / Total (percent)
	Mild-Dpy Mild-GFP Non-Roller	Dpy Non-GFP Non-Roller	Non-Dpy Bright-GFP Roller	Dpy GFP Non-Roller	Mild-Dpy Bright-GFP Non-Roller	Non-Dpy Non-GFP	Mild-GFP Roller	Non-Dpy Mild-GFP Non-Roller	
WT	1726 (54.4)	600 (18.9)	847 (26.7)	2 (0.06)	0 (0)	0 (0)	0 (0)	0 (0)	2/3175 (0.06)
rtel-1	1533 (58.4)	410 (15.6)	639 (24.3)	35 (1.30)	0 (0)	8 (0.30)	0 (0)	0 (0)	43/2625 (1.6)
lig-4	897 (58.8)	219 (14.4)	401 (26.3)	7 (0.46)	0 (0)	1 (0.07)	0 (0)	0 (0)	8/1525 (0.52)
polq-1	1315 (58.0)	336 (14.8)	608 (26.8)	8 (0.35)	0 (0)	0 (0)	0 (0)	0 (0)	8/2267 (0.35)
him-6	467 (49.2)	116 (12.2)	303 (31.9)	63 (6.60)	0 (0)	0 (0)	0 (0)	0 (0)	63/949 (6.6)
rcq-5	1776 (54.3)	623 (19)	867 (26.5)	0 (0)	0 (0)	2 (0.06)	0 (0)	0 (0)	2/3268 (0.06)
dog-1	1259 (57)	415 (18.8)	537 (24.2)	1 (0.05)	0 (0)	0 (0)	0 (0)	0 (0)	1/2212 (0.05)
brc-1	1147 (59.6)	435 (22.6)	306 (15.9)	31 (1.60)	0 (0)	0 (0)	6 (0.31)	0 (0)	37/1925 (1.9)
msh-2	1178 (54.4)	372 (17.7)	612 (28.3)	3 (0.14)	0 (0)	0 (0)	1 (0.05)	0 (0)	4/2166 (0.18)
msh-6	1040 (55)	384 (20.3)	462 (24.4)	5 (0.27)	0 (0)	1 (0.53)	0 (0)	0 (0)	6/1892 (0.32)
mlh-1	696 (55.1)	263 (20.8)	301 (23.9)	3 (0.24)	1 (0.08)	0 (0)	0 (0)	0 (0)	4/1264 (0.32)
pms-2	600 (56.6)	171 (16.1)	287 (27)	3 (0.28)	0 (0)	0 (0)	0 (0)	0 (0)	3/1061 (0.28)
rtel-1; lig-4	652 (57.1)	162 (14.2)	296 (25.9)	31 (2.7)	0 (0)	0 (0)	0 (0)	0 (0)	31/1141 (2.7)
rtel-1; polq-1	241 (60.25)	64 (16)	83 (20.75)	12 (3.0)	0 (0)	0 (0)	0 (0)	0 (0)	12/400 (3.0)
msh-2 rtel-1	673 (51.3)	312 (23.8)	322 (24.6)	4 (0.30)	0 (0)	0 (0)	0 (0)	0 (0)	4/1311 (0.31)
msh-2; him-6	240 (59.7)	30 (7.5)	98 (24.4)	29 (7.20)	2 (0.5)	0 (0)	3 (0.75)	0 (0)	34/402 (8.46)
msh-2; brc-1	1029 (54.2)	405 (21.3)	463 (24.4)	1 (0.05)	1 (0.05)	0 (0)	0 (0)	0 (0)	2/1899 (0.11)
msh-6 rtel-1	543 (57)	157 (16.5)	247 (26)	4 (0.42)	1 (0.10)	0 (0)	1 (0.10)	0 (0)	6/953 (0.63)
rtel-1; mlh-1	196 (55.8)	60 (17.1)	73 (20.8)	22 (6.28)	0 (0)	0 (0)	0 (0)	0 (0)	6/351 (6.27)
mlh-1; him-6	329 (56.5)	59 (10)	119 (20.5)	54 (9.5)	6 (1)	0 (0)	0 (0)	3 (0.5)	73/580 (12.6)
rtel-1; pms-2	470 (54)	161 (18.5)	227 (26.1)	12 (1.4)	0 (0)	0 (0)	0 (0)	0 (0)	12/870 (1.4)

Reciprocal exchange events

Supplemental Table S1. Scoring heterologous recombination. Related to Figures 1, 2 and 3.

Numbers of non-recombinant and recombinant progeny of each phenotype in the indicated genetic backgrounds.
